# Genetic variants associated mRNA stability in lung

**DOI:** 10.1186/s12864-022-08405-y

**Published:** 2022-03-11

**Authors:** Jian-Rong Li, Mabel Tang, Yafang Li, Christopher I. Amos, Chao Cheng

**Affiliations:** 1 Department of Medicine, Baylor College of Medicine, Houston, TX USA; 2 Institute for Clinical and Translational Research, Baylor College of Medicine, Houston, TX USA; 3 Department of BioSciences, Biochemistry and Cell Biology, Rice University, Houston, TX USA; 4 Dan L Duncan Comprehensive Cancer Center, Baylor College of Medicine, Houston, TX USA

**Keywords:** Expression quantitative trait loci (eQTLs), RNA-Seq, mRNA stability, Stability QTLs (stQTLs)

## Abstract

**Background:**

Expression quantitative trait loci (eQTLs) analyses have been widely used to identify genetic variants associated with gene expression levels to understand what molecular mechanisms underlie genetic traits. The resultant eQTLs might affect the expression of associated genes through transcriptional or post-transcriptional regulation. In this study, we attempt to distinguish these two types of regulation by identifying genetic variants associated with mRNA stability of genes (stQTLs).

**Results:**

Here, we presented a computational framework that takes advantage of recently developed methods to infer the mRNA stability of genes based on RNA-seq data and performed association analysis to identify stQTLs. Using the Genotype-Tissue Expression (GTEx) lung RNA-Seq data, we identified a total of 142,801 stQTLs for 3942 genes and 186,132 eQTLs for 4751 genes from 15,122,700 genetic variants for 13,476 genes on the autosomes, respectively. Interestingly, our results indicated that stQTLs were enriched in the CDS and 3’UTR regions, while eQTLs are enriched in the CDS, 3’UTR, 5’UTR, and upstream regions. We also found that stQTLs are more likely than eQTLs to overlap with RNA binding protein (RBP) and microRNA (miRNA) binding sites. Our analyses demonstrate that simultaneous identification of stQTLs and eQTLs can provide more mechanistic insight on the association between genetic variants and gene expression levels.

**Supplementary Information:**

The online version contains supplementary material available at 10.1186/s12864-022-08405-y.

## Background

Quantitative trait loci (QTLs) are genomic loci that explain variation of a quantitative trait [1]. The most well investigated QTLs are eQTLs, which are associated with the expression level of gene transcripts [2]. Assuming different regulatory mechanisms, eQTLs proximal to and distant from the transcription start site (TSS) of genes are called *cis*-eQTLs (< 1 Mb) and *trans*-eQTLs (> 5 Mb), respectively [3]. By combining high-throughput gene expression data, genetic variant profiles, and phenotype information, eQTLs can be identified systematically using a GWAS (genome-wide association study) approach [4]. It has been shown that genetic variants (single nucleotide polymorphisms) associated with complex traits, including human diseases, are more likely to be eQTLs [5]. Genetic variants located in *cis*-regulatory elements (CREs), in particular, can influence the expression of targeted genes. Indeed, eQTLs are associated with many classes of CREs that are enriched in promoters, enhancers, insulators, transcription factor (TF) binding sites, and DNase hypersensitive sites (DHSs) [6–10].

Gene expression level is regulated at both the transcriptional and post-transcriptional levels. At the transcriptional level, TFs regulate the transcription rate of genes by interacting with their promoters and enhancers [11, 12]. TF binding and histone modification signals in the TSS proximal regions account for over 50% of variation of gene expression [13–15]. Genetic variants with functional impacts on TF binding motifs or promoter/enhancer accessibility are also expected to have effects on the transcription rate of related genes [16, 17]. On the other hand, at the post-transcriptional level, mRNA stability is under intensive regulation by microRNAs and RNA-binding proteins (RBPs) [18, 19]. Genetic variants can also affect mRNA stability by interacting with microRNAs or RBPs. For example, the allele T of rs907091 located in the 3’UTR of *IZKF3* confers a miR-326 binding site, which leads to decreased mRNA stability and down-regulation of the gene; however, this is not seen with the allele C [20]. Additionally, some intronic genetic variants may also affect gene expression by interacting with splicing factors or other types of RBPs [21]. Therefore, it is often difficult to precisely interpret the eQTLs identified from high-throughput analysis. Namely, for many eQTLs, it is difficult to determine whether they influence gene expression through affecting transcriptional rate or mRNA stability. This problem is further complicated by linkage disequilibrium (LD) between neighboring genetic variants. Although high-throughput technologies that measure mRNA decay rates have been developed [22–24], there are no QTL studies that identify genetic variants associated with mRNA stability due to a lack of matched stability and genotype data.

In many eQTL studies, gene expression was determined using exons aligned reads generated by RNA sequencing (RNA-Seq) experiments [25–27]. While the protocol was designed to generate cDNA fragments from mature mRNAs, there was also a significant proportion of reads captured from intronic sequences in RNA-seq data [25]. Several studies proposed that the intronic reads of RNA-Seq were related to nascent transcription and transcriptional activity [27–30]. Based on this concept, computational methods have been developed to calculate mRNA stability based on RNA-seq data [27, 31, 32]. One method, proposed by Gaidatzis et al. [27], is called exon-intron split analysis (EISA) and discriminates transcriptional and post-transcriptional regulation of gene expression. Given the RNA-seq data in two experiment conditions, EISA calculates changes in reads mapped to exons (Δexon) and introns (Δintron) for each gene. It was shown that Δexon-Δintron was significantly correlated with experimentally measured mRNA stability changes between ESCs and terminal neurons [27]. The EISA method was then further improved and implemented in a software package, REMBRANDTS, to measure the stability of mRNAs more accurately [32].

Motivated by these methods, we developed a framework to simultaneously identify genetic variants associated with gene expression (eQTL) or mRNA stability (stQTL). We then applied this framework to the lung tissue RNA-Seq data generated by the Genotype-Tissue Expression (GTEx) project [33, 34] as a demonstration. For this data, we estimated mRNA stability using REMBRANDTS and gene expression, and then performed association analysis to 15,122,700 genetic variants for 13,476 autosomal genes. Next, we identified a total of 186,132 eQTLs for 4751 genes and 142,801 stQTLs for 3942 genes. From our analysis, we found that both stQTLs and eQTLs are enriched in the 3’UTR and CDS regions while eQTLs are also enriched in the 5’UTR and upstream region of TSS. Compared to eQTLs, stQTLs more frequently overlapped with the binding sites of RBPs and miRNAs. To explore the role of stQTLs in mRNA stability, we took a few examples to investigate the effect of genetic variants on the binding of RBPs or TFs. Together, these results suggested that the simultaneous identification of stQTLs and eQTLs can provide a useful method to better understand the molecular mechanisms underlying genetic variants.

## Results

### Overview of this study

**Fig. 1:**
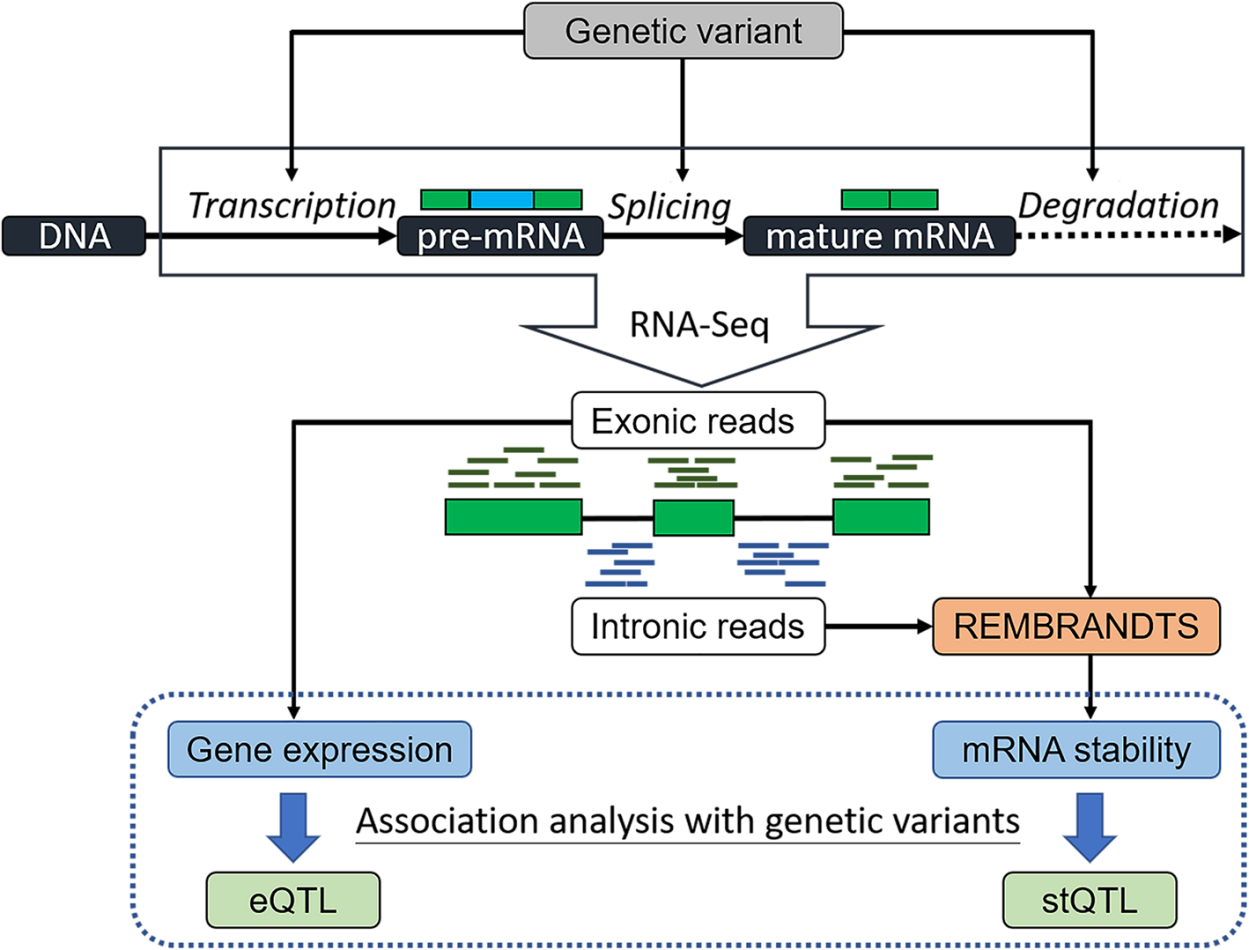
The workflow for identification of stQTLs and eQTLs using RNA-Seq. A genetic variant may regulate gene expression by affecting transcription, splicing, or stability at different stages of the life cycle of an mRNA. Both gene expression and mRNA stability can be estimated from RNA-Seq. Therefore, both expression quantitative trait loci (eQTLs) and stability quantitative trait loci (stQTLs) can be identified with genetic variations using the association analysis. By comparing stQTLs and eQTLs, it is possible to distinguish the regulatory mechanisms underlying an eQTL

Figure 1 shows the rationale underlying this study. During gene expression, a gene is transcribed into a pre-mRNA, after which the introns are removed while the exons are connected into the mature mRNA. The mature mRNA is under post-transcriptional regulation by miRNAs and other mechanisms. As shown, genetic variants can not only regulate mRNA splicing but also regulate gene expression-related traits by affecting transcription rate or mRNA stability (stability QTL, denoted as stQTL hereafter). From RNA-seq data, we can determine the reads mapped to exonic regions to obtain gene expression levels. The mRNA stability can also be calculated by combining the reads aligned to exonic and intronic regions using the REMBRANDTS [32] algorithm. Genetic variants associated with gene expression are then identified to obtain eQTLs. As gene expression is controlled by both transcription rate and mRNA stability, eQTLs are actually a mixture of QTLs affecting transcription and stQTLs. Performing an association analysis of gene expression or stability on genetic variation can identify eQTLs and stQTLs, respectively. Simultaneous identification of eQTLs and stQTLs can provide a higher resolution to understand how genetic variants affect gene expression and will allow us to infer whether a genetic variant regulates gene expression by affecting transcription activity or RNA stability. As a proof-of-concept, in this study, we applied this framework to GTEx data to simultaneously investigate the eQTLs and stQTLs in lung tissue as a demonstration.

### Expression QTLs and stability QTLs of human lung tissue

To identify and explore stQTLs and eQTLs, we processed the raw RNA-seq data for lung tissues generated by the GTEx project [34]. After performing quality trimming, alignment, and replicate merging from the same donors, we obtained the expression profiles of genes for a total of 289 subjects with matched genetic variation data. With REMBRANDTS, for each subject, we calculated the relative mRNA stability for 13,476 genes with intronic regions and constitutive exons. For QTL identification, we performed the association analysis on 15,122,700 variant-gene pairs, including 7,014,861 variants located within 100Kb upstream of TSS and 100Kb downstream of TTS for 13,476 genes, using gene expression or mRNA stability as traits. We determined a distance of 100Kb instead of 1 Mb to focus on comparing eQTLs and stQTLs since SNPs that affect RNA stability are theoretically more likely to be located near the transcription region. We identified a total of 142,801 stQTLs (Additional file 1 Table S1) and 186,132 eQTLs (Additional file 2 Table S2) at the significance level of FDR < 5%. It should be noted that here we applied a low REMBRANDTS stringency (Table S1) in order to calculate the stability for a large number of genes. A higher stringency indicates that the stability of the gene was estimated based on a larger number of reads and therefore associated with a higher confidence level. The numbers of QTLs were summarized in Table 1 according to the location of variants on each QTL’s corresponding genes.

**Table 1:** The summary of the stQTLs and eQTLs identification in GTEx lung tissue samples

Location	Number of stQTL (%)	Number of eQTL (%)	Number of variant-gene	Average length
Upstream	50,109 (35.09%)	73,647 (39.57%)	5,591,610	99,984.82
5’UTR	3877 (2.71%)	5802 (3.12%)	350,293	204.94
CDS	3949 (2.77%)	4404 (2.37%)	143,915	1148.71
Intron	32,675 (22.88%)	37,232 (20.00%)	3,281,521	50,230.12
3’UTR	2506 (1.75%)	2411 (1.30%)	123,856	727.48
Downstream	49,685 (34.79%)	62,636 (33.65%)	5,631,505	99,960.72
Total	142,801 (100.00%)	186,132 (100.00%)	15,122,700	252,256.79

The location indicates the genic position of the genetic variant in its corresponding gene in a QTL. The percentage was calculated from the number of QTLs at each location divided by the total number of QTLs

**Fig. 2:**
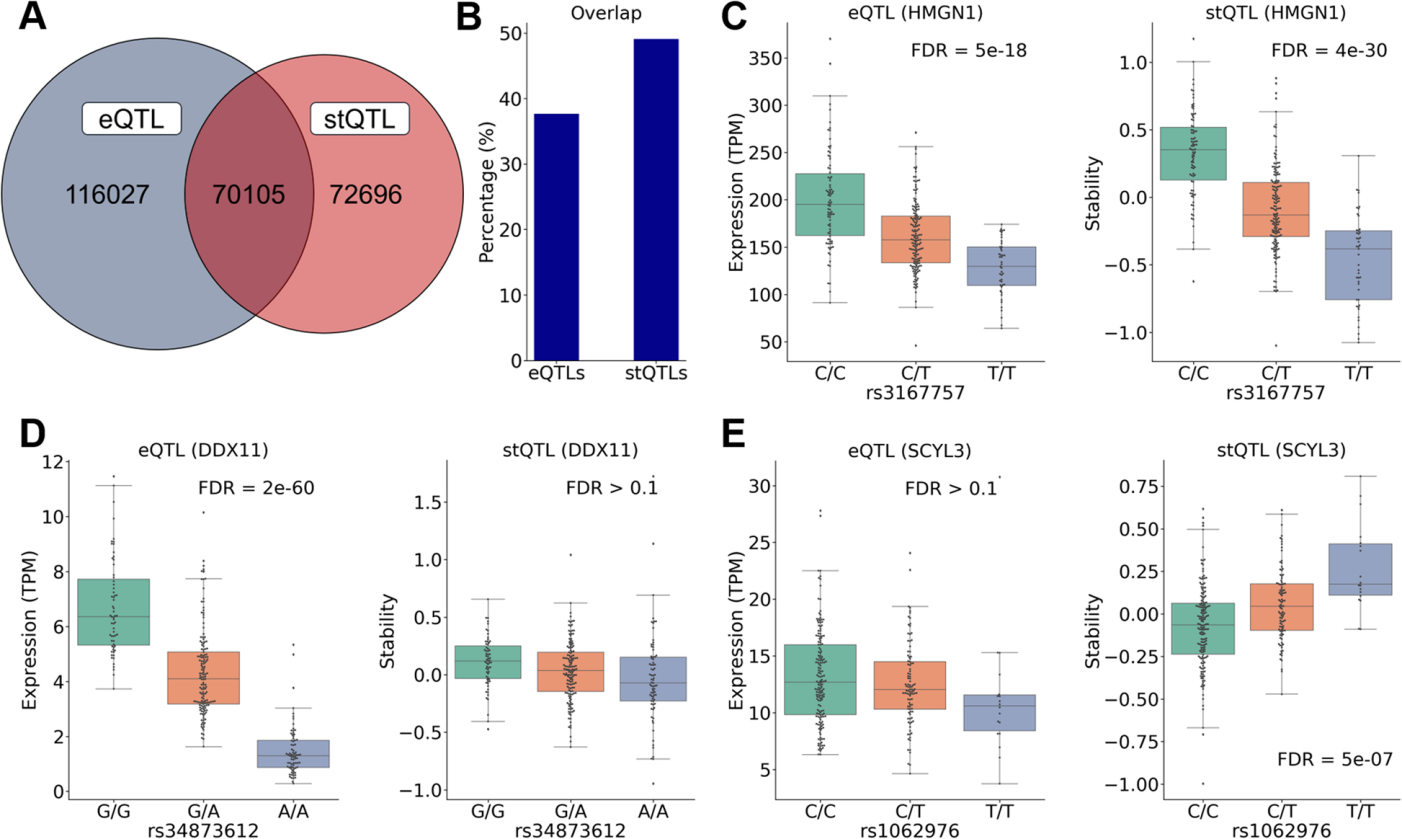
The simultaneous identification of stQTLs and eQTLs using GTEx lung tissue samples shows highly overlapped QTLs and provides additional information for investigating regulatory effects of genetic variants. **A** The Venn diagram between eQTLs and stQTLs shows that 70,105 genetic variants are both eQTLs and stQTLs. **B** The bar plot shows the percentage of overlapped QTLs in stQTLs and eQTLs, respectively. **C***HMGN1*-rs3167757 is an eQTL and a stQTL. The expression level and RNA stability of *HMGN1* will decrease as rs3167757 changes with CC > CT/TT. The rs3167757 is located on the binding sites of several RBPs in the 3’UTR region of *HMGN1*. **D***DDX11*-rs34873612 is an eQTL but not an stQTL. The expression level, but not RNA stability, of the *DDX11* will decrease as rs34873612 changes with GG > GA/AA. The rs34873612 overlaps the binding sites of several TFs in the 5’UTR region of *DDX11*. **E***SCYL3*-rs1062976 is an stQTL but not an eQTL. The RNA stability of the *SCYL3* will be affected by rs1062976, which is located in the 3’UTR region of *SCYL3*. The allele T of rs1062976 disrupts the binding motif of PTBP1 (destabilizer) but confers the binding motif of YBX1 (stabilizer)

Ideally, we would expect that all stQTLs are also eQTLs since a genetic variant that regulates RNA stability should also affect gene expression. However, in practice, the identification of different QTL types is complicated by multiple factors, including differential statistical power and LD between genetic variants. Nevertheless, we still observed that there is a very high proportion (70,105) of overlap between stQTLs and eQTLs (Fig. 2A). Out of these variants, 31,544 (45.0%) with alternate alleles associated with higher gene expression were also associated with higher stability, and 36,278 (51.7%) with alternate alleles associated with lower expression were also associated with lower stability. Indeed, 96.7% of overlapped stQTLs/eQTLs have the same effect direction on both gene expression and RNA stability, indicating that at least some of these eQTLs affect gene expression through regulating mRNA stability. We also found that 49% of stQTLs were also eQTLs (Fig. 2B), suggesting that nearly half of stQTLs do also significantly affect gene expression. On the contrary, only 37% of eQTLs were also stQTLs. This indicated that although a considerable part of eQTLs were derived from genetic variants that significantly affect stability, more of them were regulated by genetic variants affecting other factors related to gene expression.

By investigating stQTLs and eQTLs together, it is possible to determine the regulatory mechanisms underlying an eQTL. For example, genetic variant rs3167757 is significantly associated with *HMGN1* expression level (eQTL, FDR = 5e-18) with CC > CT/TT (Fig. 2C). As shown, this genetic variant is also associated with *HMGN1*’s mRNA stability (stQTL, FDR = 3.7e-30). This result indicated that rs3167757 might regulate the expression level of *HMGN1* by affecting its mRNA stability. Indeed, *HMGN1*-rs3167757 has also been reported as an eQTL in lymphoblastoid cell lines (LCLs) [35, 36]. The rs3167757 is located at the 3’UTR of the *HMGN1* gene and overlaps with binding sites of 20 different RBPs [37]. According to the analysis using RBPmap [38] (Additional file 3 Table S3), while the allele C of rs3167757 confers a motif for eight RBPs (CUG-BP, HNRNPF, MBNL1, SFPQ, TRA2B, HNRNPL, SRSF3, and YBX2), the allele T disrupts the binding motifs of five of the RBPs (HNRNPF, MBNL1, SFPQ, TRA2B, and YBX2). Notably, among them, HNRNPF [39, 40], MBNL1 [41, 42], and YBX2 [43] are known to contribute to mRNA stabilization. This is consistent with the observation that genotype CC is associated with higher stability of *HMGN1* mRNA than CT and TT. As another example, genetic variant rs34873612 is significantly associated with *DDX11* expression level (eQTL, FDR = 2e-60) but not with *DDX11* mRNA stability (FDR > 0.1) with GG > GA/AA (Fig. 2D). This result suggested that rs34873612 might regulate the expression level of *DDX11* by affecting the transcription rate rather than its mRNA stability. According to the PROMO prediction [44], the rs34873612 is located at the 5’UTR of the *DDX11* gene and overlaps with the binding site of three TFs: GR-alpha, GATA2, and GATA3. While the allele G contributes to the binding motifs of these TFs, the allele A disrupts the binding motif of GATA3, which potentially contributes to the decreased *DDX11* expression seen in the GA and AA genotypes (Fig. 2D). mRNA stability only contributes partially to gene expression level; consistently, many genetic variants are found to be stQTLs but not eQTLs. For example, rs1062976 is significantly associated with the mRNA stability of *SCYL3* (stQTL, FDR = 5e-07) but not its expression level (not an eQTL, FDR > 0.1) with CC > CT/TT (Fig. 2E). Overall, our results indicated that simultaneous identification of stQTLs and eQTLs can provide us with more detailed biological insights on the regulatory effects of genetic variants on a large scale.

### Distributions of eQTLs and stQTLs across genic regions

**Fig. 3:**
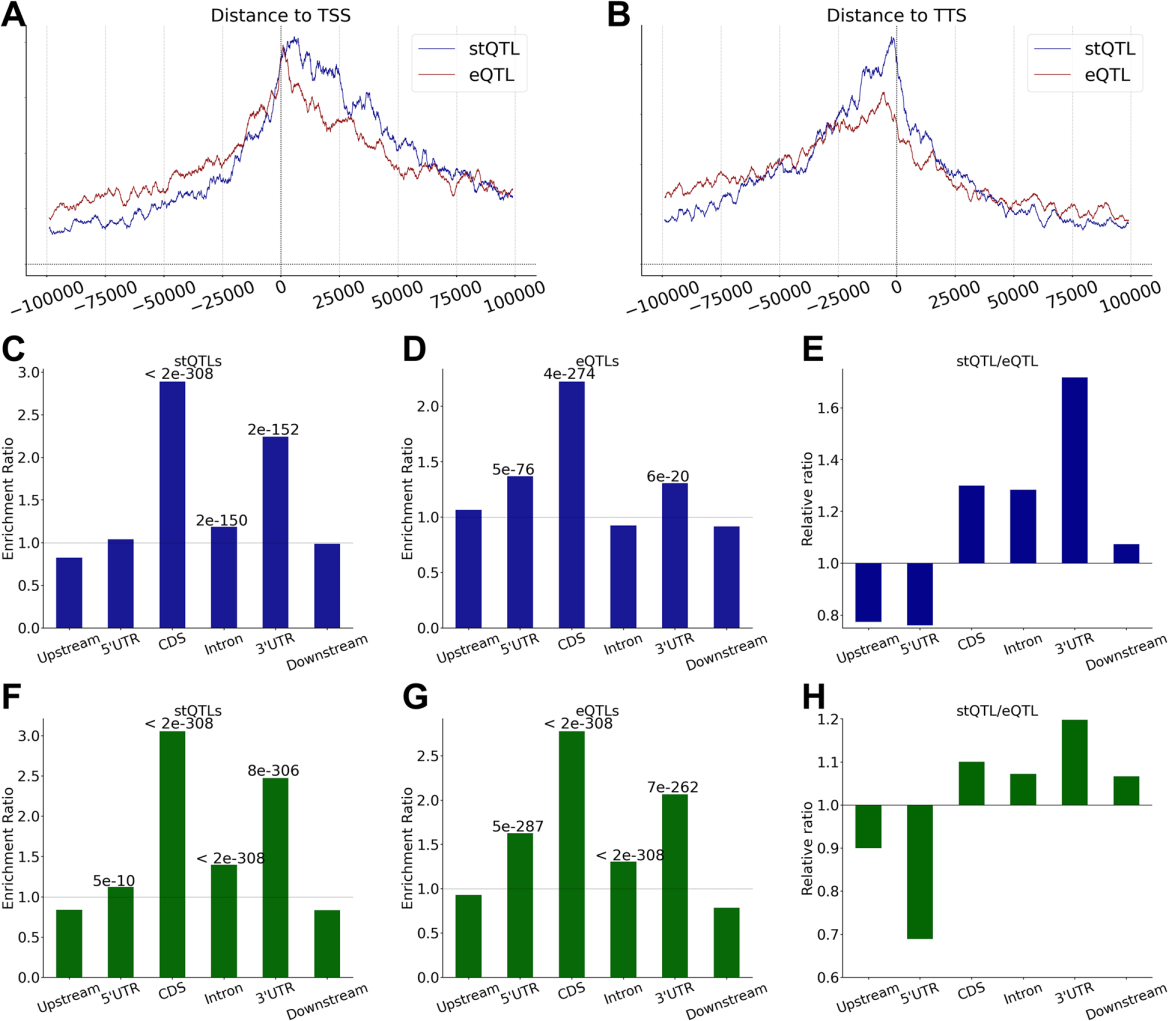
There are biased distributions in different genic regions of eQTLs and stQTLs. **A** The distribution from the enrichment ratio of stQTLs and eQTLs to TSS. Plot has a bin size of 2000 bp and a sliding window of 50 bp. **B** The distribution from the enrichment ratio of stQTLs and eQTLs to TTS. Plot has a bin size of 2000 bp and a sliding window of 50 bp. **C** The enrichment ratio in different genic locations of stQTLs before LD fine-mapping. The upstream indicates the region of 100Kb upstream from TSS, and the downstream indicates the region of 100Kb downstream from TTS. (The following figs are the same) **D** The enrichment ratio in different genic locations of eQTLs. **E** The relative proportion of enrichment ratio in different genic locations between stQTLs and eQTLs. **F** The enrichment ratio in different genic locations of stQTLs after LD fine-mapping. **G** The enrichment ratio in different genic locations of eQTLs after LD fine-mapping. **H** The relative proportion of enrichment ratio in different genic locations between stQTLs and eQTLs after LD fine-mapping

stQTLs are associated with mRNA stability while eQTLs are associated with gene expression by affecting either mRNA stability or gene transcription. Therefore, we expect their distributions in genes to differ. To examine this, we looked at the distribution of eQTLs and stQTLs in the DNA regions surrounding TSS and TTS of genes. We found that eQTLs are more likely to be located upstream of TSS of their corresponding genes while stQTLs tend to be located downstream of TSSs (Fig. 3A). On the other hand, stQTLs are more likely to be located in the region from TTS to 10Kb upstream than eQTLs. Both stQTLs and eQTLs are more likely to be located in the upstream region of TTS rather than the genes’ downstream regions (Fig. 3B). Subsequently, we divided genic regions associated with genes into upstream, 5’UTR, CDS, Intron, 3’UTR, and downstream regions and then examined the distributions of eQTLs and stQTLs in these regions. Using the distributions of all genetic variants as the background, we calculated the enrichment ratio of stQTLs and eQTLs by using a hypergeometric test [45]. As shown in Fig. 3C, stQTLs are enriched by 2.89-fold in the CDS (*P* < 2e-308) and by 2.25-fold in 3’UTR (*P* = 2e-152) regions. This result is consistent with the fact that genetic variants located in these regions may have functional impacts on mRNA stability by affecting RNA secondary/tertiary structure or RBP/microRNA binding. stQTLs are also slightly enriched in intron regions (ER = 1.19 and P = 2e-150). In contrast, eQTLs are enriched in the CDS (ER = 2.22, *P* = 4e-274), upstream (ER = 1.10, *P* = 3e-65), 5’UTR (ER = 1.37, *P* = 5e-76), and 3’UTR (ER = 1.30, *P* = 6e-20, Fig. 3D) regions, respectively. The enrichment of eQTLs in these regions may be explained by how gene expression can be determined not only by transcriptional activity (genetic variants in upstream, 5’UTR, or CDS regions) but also by RNA stability (genetic variants in CDS or 3’UTR regions). We compared the enrichment ratios of stQTLs and eQTLs and found that stQTLs are more likely to be in the CDS, intron, and 3’UTR regions, while eQTLs are enriched in the upstream and 5’UTR regions (Fig. 3E).

It should be noted that the resolution of QTL analysis is affected by linkage disequilibrium (LD) between neighboring genetic variants. Based on the genotype data for lung samples used in this study, we performed LD analysis and observed that many eQTL/stQTL loci were in high LD (r^2^ > 0.9) with each other (Additional file 3 Fig. S1A and S1B). Furthermore, we performed GARFIELD [46] to adjust the LD confounding to investigate the enrichment of stQTL/eQTL in histone modifications (Additional file 3 Table S4), and we found that while both QTLs are depleted in the H3K27me3 (marks of polycomb repression), eQTLs are slightly more so compared to stQTLs. In addition, stQTLs are more enriched in H3K36me3 (marks of transcribed regions, odds ratio = 1.53, *P* = 1e-262) and H4K20me1 (enriched within transcribing gene bodies [47], odds ratio = 1.21, *P* = 3e-19) than eQTLs.

To best exclude the influence of LD on the genic distribution, we performed another association analysis that added 40 PEER factors (probabilistic estimation of expression residuals) [48] generated by the PEER package [49, 50]. The CAVIAR [51] was used as the fine-mapping step to define the 90% credible causal variants sets to identify a total of 108,412 stQTLs and 163,929 eQTLs (Additional file 4 Table S5 and Additional file 5 Table S6). We investigated the genic distribution of the fine-mapped stQTL/eQTL and found both were more enriched in the 5’UTR, CDS, intron, and 3’UTR regions than before LD analysis (Fig. 3F and G). When the distributions of causal stQTLs and eQTLs were directly compared, eQTLs were more likely to be in the upstream and especially 5’UTR regions than stQTLs (Fig. 3H), while stQTLs were more enriched than eQTLs in the CDS and 3’UTR regions. Additionally, we investigated the distribution of the stQTL/eQTL with LD filtering that selected the most significant variant as the representative stQTL/eQTL within each determined LD block (r^2^ > 0.9), and we found that the distribution after LD filtering (Additional file 3 Fig. S2A and S2B) is consistent with that after fine-mapping (Fig. 3F and G). Similarly, stQTLs were also more likely to be in the CDS and 3’UTR regions than eQTLs (Additional file 3 Fig. S2C). The results suggested that the distribution of stQTLs and eQTLs differs possibly due to their potential biological functional differences.

### stQTLs are significantly enriched in RBP binding sites

**Fig. 4:**
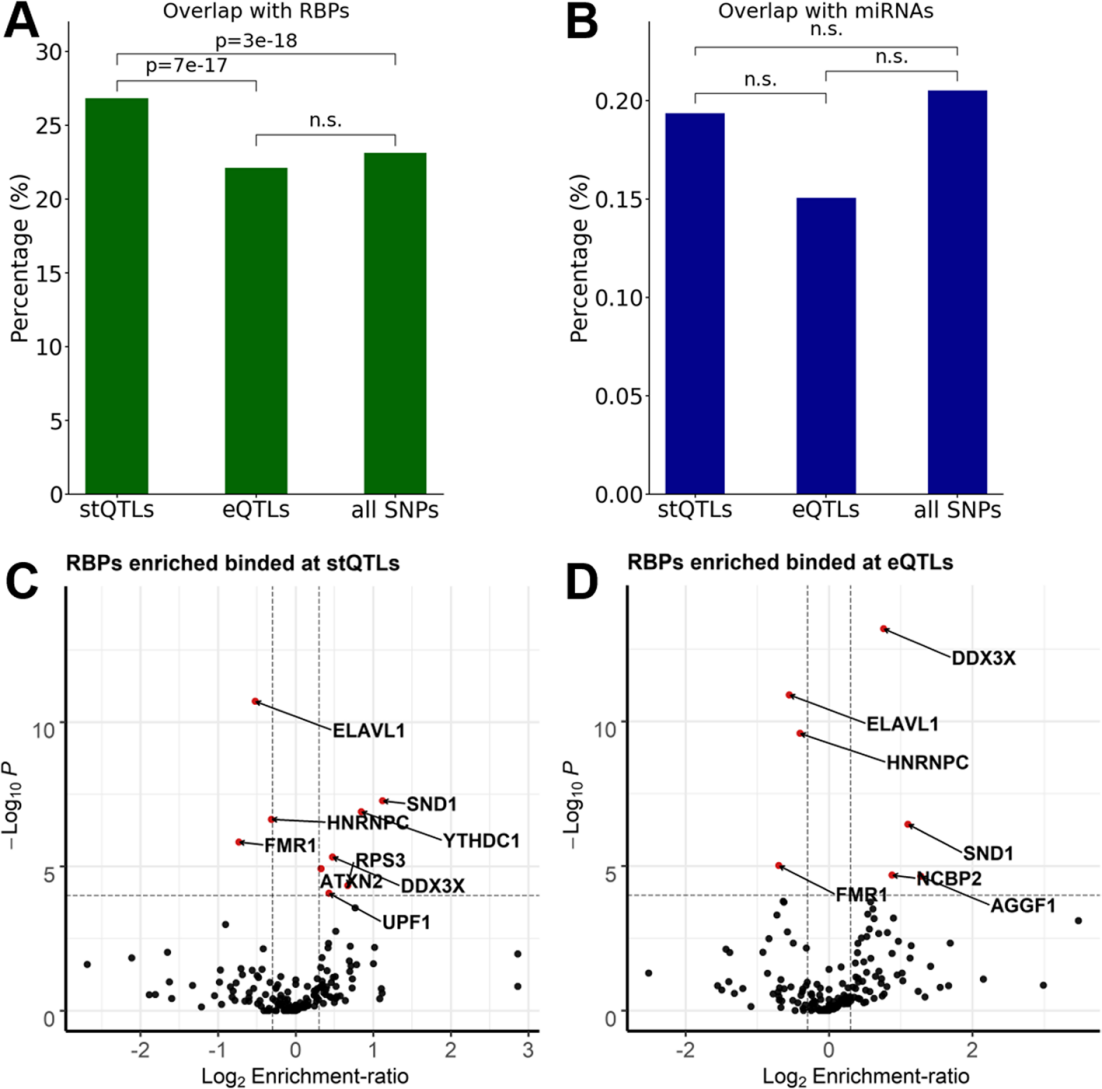
Enrichment of stQTLs and eQTLs in the binding sites of RBPs and miRNAs. **A** Proportion of overlap between stQTLs, eQTLs, and all genetic variants and RBP binding sites in mature mRNA. The statistical significance was calculated using Fisher’s exact test. The n.s. indicates not significant. **B** Proportion of overlap between stQTLs, eQTLs, and all genetic variants and miRNA binding sites in mature mRNA. The statistical significance was calculated using Fisher’s exact test. The n.s. indicates not significant. **C** The volcano plot shows nine RBPs (red points) whose binding sites were significantly (−Log_10_*p*-value > 4, 2-sides Fisher’s exact test) enriched (six RBPs, Log_2_ Enrichment-ratio > 0.3) or depleted (three RBPs, Log_2_ Enrichment-ratio < −0.3) in stQTLs. **D** The volcano plot shows seven RBPs (red points) whose binding sites were significantly (−Log_10_ p-value > 4, 2-sides Fisher’s exact test) enriched (four RBPs, Log_2_ Enrichment-ratio > 0.3) or depleted (three RBPs, Log_2_ Enrichment-ratio < − 0.3) in eQTLs

After demonstrating the enrichment of stQTLs in the 3’UTR and CDS regions, we then examined whether stQTLs tend to locate in the binding sites of RBPs or miRNAs, many of which are known to be involved in post-transcriptional regulation of mRNAs. To this end, we investigated the binding sites of RBPs and miRNAs provided by Postar2 [37] and TargetScan [52], respectively, to annotate the stQTLs identified in our analysis. Our results indicated that stQTLs (*P* = 3e-18, Fisher’s exact test) but not eQTLs (*P* > 0.1, Fisher’s exact test) are enriched in RBP binding sites. In fact, we found that 26.81% (2770/10,332) of stQTLs overlap with the binding sites of at least one RBP, which is significantly higher (*P* = 7e-17, Fisher’s exact test) than 22.10% (2788/12,617) for eQTLs (Fig. 4A). In addition, we have also examined the overlap with miRNA binding sites and observed a higher proportion of stQTLs (0.19%, 20/10,332) than eQTLs (0.15%, 19/12,617) in the miRNA binding sites, although no statistical significance was detected due to very small genomic regions covered by miRNA binding sites (Fig. 4B). To investigate whether the overlap between stQTLs and RBP binding sites was a coincidence, we examined the enrichment for stQTLs to all variant-gene pairs in overall RBP binding sites in the 5’UTR, CDS, Intron, and 3’UTR regions, separately (Additional file 3 Table S7). Compared with the variants located in RBP binding sites, stQTLs were enriched by about 2.47-fold (*P* < 2e-308, hypergeometric test) in the CDS region and by 2.31-fold (P < 2e-308) in the 3’UTR region, but not in the 5’UTR (ER = 0.79, P > 0.1) and Intron regions (ER = 0.71, P > 0.1). This result indicated that stQTLs were likely not coincidentally overlapped with the RBP binding sites but have a potential biological function relationship with RBPs.

We also performed Fisher’s exact test to identify RBPs whose binding sites were enriched for stQTLs (Fig. 4C and Additional file 3 Table S8) or eQTLs (Fig. 4D and Additional file 3 Table S9). We identified a total of six significant RBPs (*P* < 1e-04) including SND1, YTHDC1, DDX3X, ATXN2, RPS3, and UPF1 (as shown in Table 2). Interestingly, SND1 [53–55], DDX3X [56, 57], ATXN2 [58, 59], and RPS3 [60] were known to stabilize their bound mRNAs, while UPF1 is the key factor of the nonsense-mediated mRNA decay pathway [61–63]. Moreover, YTHDC1 is a well-known m^6^A (*N*^6^-Methyladenosine) reader [64], which has been found to regulate mRNA splicing [65, 66], alternative polyadenylation [66], and stability [67, 68] through recognizing m^6^A. Similarly, we identified four RBPs whose binding sites were significantly enriched for eQTLs (P < 1e-04, Table 3), among which the two most significant RBPs, DDX3X and SND1, were also enriched for stQTLs. Of the other RBPs whose binding sites were significantly enriched with eQTLs, NCBP3 can regulate gene expression by forming a cap binding complex that binds to the 5’cap of pre-mRNA to promote splicing, 3′-end processing, and mRNA exporting [69–71] while AGGF1 was found to repress the expression of pro-inflammatory molecules [72]. Although RBP regulation is affected by many factors such as the subcellular localization and RNA substrates [73, 74], which makes it a challenging task to systematically investigate the degree of consistency between the regulation of RBPs and QTLs, our results indicate that stQTLs or eQTLs located in the RBPs binding sites are likely to have regulations on RNA stability or expression by affecting the RBP binding.

**Table 2:** RBPs whose binding sites were enriched for stQTLs

RBPs	stQTL	non-stQTL	ER	*p*-value	FDR
SND1	64	1504	2.17	4E-08	5E-06
YTHDC1	100	2850	1.80	1E-07	7E-06
DDX3X	226	8348	1.39	3E-06	1E-04
ATXN2	436	17,911	1.25	7E-06	3E-04
RPS3	91	2923	1.59	3E-05	0.001
UPF1	198	7533	1.35	5E-05	0.002

Six RBPs significantly overlap (Log2 Enrichment-ratio > 0.3 and *p*-value <1e-04, Fisher’s exact test) with stQTLs in mature mRNAs in lung. ER: Enrichment ratio

**Table 3:** RBPs whose binding sites were enriched for eQTLs

RBPs	eQTL	non-eQTL	ER	*p*-value	FDR
DDX3X	250	8331	1.69	5E-14	1E-11
SND1	58	1509	2.15	2E-07	2E-05
NCBP2	60	1819	1.84	1E-05	6E-04
AGGF1	30	689	2.43	2E-05	6E-04

Four RBPs significantly overlap (Log2 Enrichment ratio > 0.3 and *p*-value <1e-04, Fisher’s exact test) with eQTLs in mature mRNAs in lung. ER: Enrichment ratio

### Sex-specific stQTLs

**Fig. 5:**
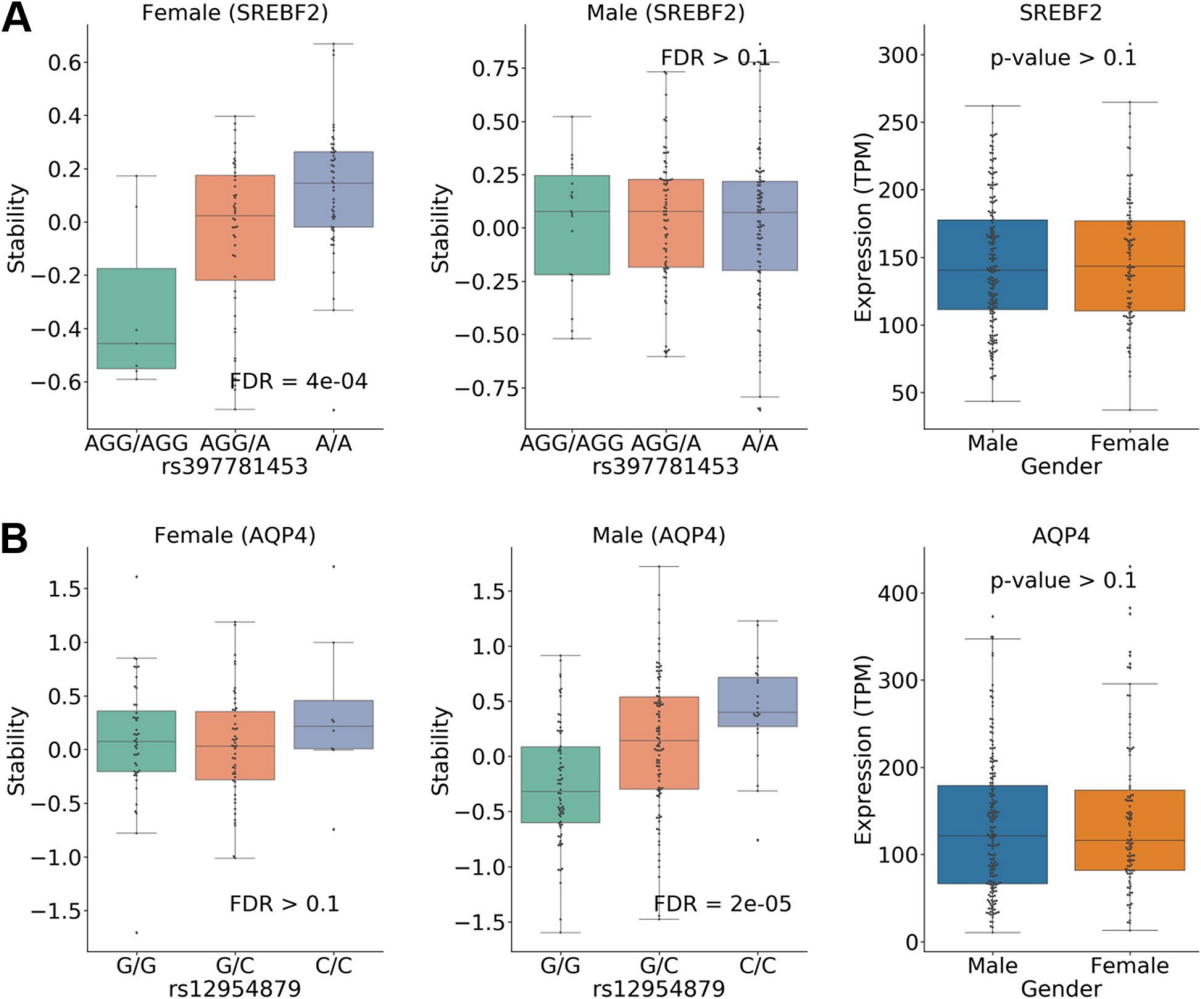
Sex-specific stQTLs identification. **A** The association between genetic variant rs397781453 and the RNA stability of *SREBP2* is female-specific (stQTL, FDR = 4e-04), but this pattern does not occur in males (FDR ≥ 0.1). In the right panel, the expression of *SREBP2* is not significantly different (*P* ≥ 0.1) between male and female samples. **B** The association between genetic variant rs12954879 and the RNA stability of *AQP4* is male-specific (stQTL, FDR = 2e-05), but this pattern does not occur in females (FDR ≥ 0.1). In the right panel, the expression of *AQP4* is not significantly different (P ≥ 0.1) between male and female samples

Finally, we examined whether some genetic variants were associated with mRNA stability in a sex-specific manner and denoted them as sex-specific stQTLs. We divided 289 samples into 187 males and 102 females, and then performed association analysis with covariates to implement the Sex-specific stQTL classification. If a gene is specifically expressed in males or females, then an stQTL identification can only be performed in the corresponding sex. Therefore, we focused our analysis on 13,116 autosomal genes that are not differentially expressed (FDR > 0.05, t-test) between the sexes and then investigated a total of 14,987,511 variant-gene pairs that genetic variants located from 100Kb upstream to 100Kb downstream of a gene. Then, we defined male-specific QTLs as those that are significant in males (FDR < 0.05) but not significant in females (*P* > 0.1), and defined female-specific QTLs similarly. Finally, we identified 14,683 male-specific and 2280 female-specific stQTLs (Additional file 6 Table S10). Of note, there were no stQTLs identified that were significantly associated with RNA stability in both sexes but have opposite effects. As an example, the association between genetic variant rs397781453 and the RNA stability of *SREBP2* is female-specific (Fig. 5A). As shown, we detected a significant association in females with FDR = 4e-04 but not in males (FDR ≥ 0.1). On the other hand, the association between *AQP4* and genetic variant rs12954879 is male-specific (Fig. 5B). The RNA stability of *AQP4* is significantly associated (FDR = 2e-05) with genetic variant rs12954879 in males but not in females (FDR > 0.1). Of note, both *SREBP2* or *AQP4* have similar expression levels between males and females (the right panel of Fig. 5A and B). In addition, we performed the association analysis including a genotype-sex interaction term (G × S) to investigate sex-biased stQTLs [75] and identified 499 sex-biased stQTLs at the G × S significance level of FDR ≤ 25%. For each sex-biased stQTL, we performed sex-stratified stQTL analysis and identified 100 male-specific stQTLs, 174 female-specific stQTLs, and 198 sex-biased stQTLs that were significantly associated in both sexes with different allelic effect sizes at the significance level FDR < 5% (Additional file 6 Table S11).

## Discussion

In this study, we systematically identified stQTLs that are associated with mRNA stability in lung tissues and compared them with eQTLs using GTEx RNA-Seq data. Out of the 151,227,000 genetic variants within 100Kb upstream from TSS to 100Kb downstream from TTS of 13,476 corresponding genes, we identified a total of 186,132 eQTLs and 142,801 stQTLs. We found that stQTLs are mainly enriched in the 3’UTR and CDS regions, while eQTLs are enriched in the CDS, 5’UTR, 3’UTR, and upstream regions (Fig. 3F and G). We also found that stQTLs are significantly located in the binding sites of RBPs (Fig. 4A). Moreover, the different stQTL/eQTL variants will change the motifs to affect the bound RBPs, which then regulate RNA stability or gene expression (Fig. 2C-E). Our results suggest that stQTLs may significantly affect RNA stability, mostly because they are located in the 3’UTR [76, 77] and CDS [78, 79] regions that most often interact with other molecules. These results are consistent with previous studies, which have found that the codon usage and changes on CDS could affect mRNA stability [78, 80–82]; studies have also shown that the sequence in the 3’UTR region affected mRNA stability as well since it includes RBP binding sites [83, 84]. On the contrary, eQTLs are a group that utilizes complex mechanisms and may regulate expression levels by affecting stability [85, 86], transcriptional activity [87–89], and even the addition of a 5’cap or a polyA tail [69]. Therefore, while eQTLs largely resemble stQTLs but are less enriched at the 3′ UTR and CDS regions, eQTLs are also enriched in the 5’UTR and upstream regions where the enhancers and promoters that regulate transcriptional activity are located [90–92].

In this study, we used lung tissue data from GTEx as an example to demonstrate that identifying stQTLs with computationally inferred stability profiles may provide additional insights for the eQTL study. Although this method can theoretically be extended to RNA-Seq data for other tissues, it is worthwhile to note that this framework has obvious limitations. For example, the power of stQTL analysis is limited by the computational methods used for mRNA stability inference. Although previous studies have demonstrated that the EISA algorithm [27] and its improved REMBRANDTS package [32, 93, 94] used in this study achieve fairly high accuracy for mRNA stability evaluations, the accuracy of inferred mRNA stability may vary significantly between different genes. First, the differential expressed long noncoding RNAs (lncRNAs) [95, 96] or perturbated factors involved in intron degradation [27, 97] could cause the changes of difference in intronic read counts (Δintron) to affect the stability estimate. Adding the annotation of non-coding RNAs in the alignment of RNA-Seq may improve the accuracy of the mRNA stability inference. Second, it is difficult to accurately calculate stability for genes with low aligned read counts because the stability inference is based on the relative change of exonic and intronic reads (Δexon–Δintron) [32]. Of note, the REMBRANDTS provides a stringency parameter to filter genes with low read counts. In our study, we presented the results using a weak stringency of 0.01. At this setting, the stability for 13,429 genes were calculated, compared to 2593 genes when a default stringency (0.9) is used. By lower the stringency, we are able to calculate the stability for more genes, but the genes with smaller number of reads are generally associated with higher level of noise. Indeed, 41.88% (634/1514) of stQTLs with stringency ≥0.9 overlap with RBP binding sites, which is significantly higher (*P* = 6e-44, Fisher’s exact test) than 23.69% (1546/6527) of stQTLs with stringency ≤0.5. Finally, it should be of note that the mRNA stability calculated from RNA-Seq using REMBRANDTS is not an actual absolute value but a differential mRNA stability relative to the average of all samples for a given gene [32, 97, 98]. Due to these limitations, it may be difficult to directly compare the stQTLs identified using different tissue data. Therefore, we suggest that it is necessary to keep these limitations in mind before evaluating mRNA stability using RNA-Seq data.

The identification of stQTLs provides a higher resolution to better understand the molecular mechanism of genetic variants regulating gene expression, and an accurate estimation of mRNA stability is very important for the identification of stQTLs. Although some high-throughput technologies, such as BRIC-Seq [24, 99], have been developed to determine the decay rate of mRNA, these methods are often limited to only being used in cell culture conditions [32], and there are not enough samples available for QTLs research. Therefore, despite the limitations of computational approaches, such as SnapShot-Seq [29], EISA, and REMBRANDTS, our analysis for mRNA stability inference using RNA-seq by REMBRANDTS shows that the stQTL genic distribution and overlap with RBP binding sites is indeed consistent with biological theories. Furthermore, computer algorithms based on RNA-Seq are still under continuous development. For example, INSPEcT [97] was recently designed to calculate RNA kinetic rates based on time course RNA-seq data, or to estimate stability by calculating the difference between premature and mature RNA expression [100]. Going forward, stQTLs identified with more accurate mRNA stability profile estimations may further our understanding of how genetic variants regulate gene expression.

## Conclusion

In conclusion, we present a large-scale identification for eQTLs and stQTLs using RNA-Seq data in lung tissues. Our results demonstrate that there are differential genic distributions, as well as interactions with RBPs or TFs, between eQTLs and stQTLs. We show in this study that simultaneous identification of eQTLs and stQTLs provides more biological insights for better understanding the regulatory mechanisms underlying genetic variants associated with gene expression.

## Methods

### Collection of datasets

The genotype data and RNA-Seq data of lung tissues produced by the Genotype-Tissue Expression project [33] (release 7) were used in this study. The genotype data (11,555,102 variants for 450 European descent donors) has been filtered by GTEx Consortium with the following criteria: imputation quality (INFO score) < 0.4, minor allele frequency (MAF) < 1%, and Hardy-Weinberg Equilibrium (HWE) *p* < 10^− 6^ [34]. RNA-Seq SRA files and genetic variants data were downloaded from NCBI dbGaP [101] (Study Accession: phs000424.v7.p2), and subject phenotypes were collected from the GTExPortal (https://www.gtexportal.org/home/datasets). The data contains a total of 318 RNA-Seq runs and 404 genetic variant samples from 289 different subjects. For RNA-Seq analysis, the human reference genome and annotation were collected from Ensembl [102], version GRCh37.87. For RNA stability analysis, the annotation GTF files recording the coordinates of intronic and constitutive exonic segments of genes was generated using the shell script modified from the first step of the https://github.com/csglab/CRIES [32].

### Processing of RNA-Seq data

The 318 RNA-Seq SRAs were dumped into FASTQ files using SRA Toolkit (http://ncbi.github.io/sra-tools). The read quality and retained adapters were checked with FastQC [103]; then, the adapters and low-quality reads were trimmed using Trimmomatic v0.39 [104]. The alignment was performed using HISAT2 v2.1.0 [105] with default parameters, and the alignment files from the same subjects were then merged. Read counts of introns or exons were extracted separately using the HTSeq-count script of the HTSeq v0.12.4 [106] with the parameter --stranded = no. The RNA stability profiles for 289 subjects were estimated using the REMBRANDTS [32] with the parameter of linear method and 11 different stability stringency setting from 0.01 to 0.99. The stringency determines the minimum read count required for a gene to be selected for stability calculation. Therefore, genes analyzed with higher stringency have higher confidence in their stability estimates. The stringency of each gene in the QTL analysis results was recorded in Table S1 and S2. The TPM (transcripts per million) [107] was used as the expression unit for measuring the expression levels of 13,476 genes with stability profiles.

### Identifying QTLs by associating genetic variants with traits derived from RNA-seq data

For covariates construction, the plink [108] (version 1.90 beta, https://www.cog-genomics.org/plink/1.9/) was performed with the parameter --indep-pairwise 200,100 0.2 to prune a subset of genetic variants. PCA analysis was performed after removing strand ambiguous variants (AT/CG) and genetic variants located in the MHC region. The first three PCs were selected as covariates with sex and age. To consider potential confounders in the gene expression data, the latent covariates for gene expression profiles (13,453 genes with ≥0.1 TPM and ≥ 6 reads in ≥20% samples) were estimated using the Probabilistic Estimation of Expression Residuals (PEER) method [47] with the PEER package [48, 49]. A total of 40 PEER factors were generated and then added into the regression model together with other covariates. For *cis*-QTL identification, genetic variants that were located within 100Kb upstream from the TSS (transcription start site) to 100Kb downstream from the TTS (transcription termination site) of Ensembl annotated genes (GRCh37.87) were selected. The expression profile was then converted with log10(TPM*100 + 1), and the linear regression model was performed as the association analysis between the dosage of each genetic variant and the value of expression or stability of each gene. The Benjamini-Hochberg Procedure [109] was implemented to calculate the false discovery rate (FDR), and genetic variants with the association of FDR less than 0.05 were regarded as QTLs.

### Estimation of linkage disequilibrium effect

We performed the plink [108] to all genetic variants of 289 subjects with the parameter (−-r2 --ld-window 50 --ld-window-kb 100,000 --ld-window-r2 0.9) to estimate the linkage disequilibrium (LD) between each genetic variant. We then constructed LD blocks, in which r^2^ of LD between each genetic variant must be greater than 0.9. To reduce the influence of LD on the gene distribution of QTLs, we selected the QTLs with the lowest FDR of the association analysis in each LD block and then performed the enrichment analysis in different genic regions as demonstrated in the previous section.

### Fine-mapping analyses for QTLs

To exclude the influence of LD on the genic distribution, we used the CAVIAR (CAusal Variants Identification in Associated Regions) [50] as the fine-mapping step to identify the causal variants for each stQTL and eQTL. The CAVIAR was performed to all variants in each corresponding gene in each stQTL/eQTL using their LD profiles, and the Z-scores of the PEER factors included linear model with a threshold of ρ = 90% to identify the 90% credible causal variants set for each QTL.

### Enrichment analysis of QTLs in different genic regions

To determine whether eQTLs and stQTLs were evenly distributed in different genic regions, we performed the following analyses. Here, we use the stQTL as an example. First, we counted the number of all genetic variants in the TSS-upstream (from TSS to 100Kb upstream), 5’UTR, CDS, 3’UTR, intronic and TSS-downstream (from TTS to 100Kb downstream) regions. Let us use *N*^*k*^ to denote the number of all genetic variants in the kth region (*k* = 1, …, 6). We then counted the number of stQTLs in each of these regions and used *Q*^*k*^ to denote the number in the kth region. Third, to determine whether stQTLs are enriched in region *k*, we consider the following numbers: *Q*^*k*^, *Q*^(−*k*)^, *N*^*k*^-*Q*^*k*^, and *N*^(−*k*)^-*Q*^(−*k*)^, where (−*k*) indicates all regions other than *k*. Fisher’s exact test was then used to calculate the significance of enrichment. The enrichment analysis was performed separately for stQTLs and eQTLs.

### Enrichment analysis of QTLs in different histone modifications

To explore the distribution of stQTLs and eQTLs in the genomic functional regions, we applied the GARFIELD (GWAS Analysis of Regulatory or Functional Information Enrichment with LD correction) [46] method to investigate the enrichment of stQTL/eQTL in different histone modification peaks by adjusting confounding from LD. The *P* value profiles of association analysis were used as the input data of the GARFIELD. Combining the LD data, MAF data, TSS distance, and annotation data that was constructed by the GARFIELD, we performed the enrichment analysis on 55 Histone modification datasets (including 11 different Histone modifications) with the threshold of FDR < 0.05 for stQTLs and eQTLs. Stouffer’s Z-score method [110] was used to meta-analyze the enrichment results of the different datasets in the same histone modification type.

### Identification of QTLs located at binding sites of miRNAs or RBPs

stQTLs and eQTLs were mapped to the binding sites of RNA binding proteins (RBPs) and microRNAs (miRNAs). RBP binding site data were retrieved from Postar2 [37] (http://lulab.life.tsinghua.edu.cn/postar/). miRNA binding site data were downloaded from targetScanHuman [52] (http://www.targetscan.org/vert_72/). Both databases are based on the human genome reference version GRCh38. To match our analysis, we performed LiftOver [111] (https://genome-store.ucsc.edu/) to convert genome coordinates into GRCh37. To evaluate QTLs that were located on the binding sites of RBPs or miRNAs, we selected stQTLs or eQTLs on mature mRNA to align with the binding sites data, and then used the Fisher’s exact test [112] to identify RBPs whose binding sites were enriched located.

### Identification of sex-specific stQTLs

To assess whether genetic variants are associated with mRNA stability in a sex-specific manner, 289 samples were divided into 187 males and 102 females. The association analysis was performed in males and females separately using the stability profiles of 13,116 autosomal genes that are not differentially expressed (FDR > 0.05, t-test) between sexes. Male or female specific stQTLs are defined as variants that are significantly related to mRNA stability in one sex (FDR < 0.05) but not in another (*P* > 0.1). As a supplement, the linear model with adding the Genotype × Sex interaction terms (G × S) was also performed to identify sex-specific stQTLs [72]. The variants with a G × S significance level of FDR ≤ 25% in corresponding genes were selected, and the sex-stratified linear model was then performed using selected variant-gene pairs to identify the male or female specific stQTLs at FDR < 5%.

## Supplementary Information

**Additional file 1.** Contains additional Table S1 which contains 142,801 stQTLs identified in this study.

**Additional file 2.** Contains additional Table S2 which contains 186,132 stQTLs identified in this study.

**Additional file 3.** Contains additional tables (Table S3, S4, S7, S8, and S9) and figures (Fig. S1 and S2) related to enrichment of RBP binding sites, enrichment of histone modifications using GARFIELD, and genic distribution of stQTLs/eQTLs after LD filtering.

**Additional file 4.** Contains additional Table S5 which includes stQTLs that identified after adding PEER factors and filtered by CAVIAR.

**Additional file 5.** Contains additional Table S6 which includes eQTLs that identified after adding PEER factors and filtered by CAVIAR.

**Additional file 6.** Contains additional tables (Table S10 and S11) related to Sex-specific stQTLs identified using stratification analysis and Sex-SNP interaction terms, respectively.

## Data Availability

The data analyzed and generated in this study are publicly available data sets. This data can be obtained here: RNA-Seq data and genetic variants data used for the analyses described in this manuscript were obtained from dbGaP (phs000424.v7.p2, https://www.ncbi.nlm.nih.gov/projects/gap/cgi-bin/study.cgi?study_id=phs000424.v7.p2). The Individual phenotype data was downloaded from the GTEx Portal (https://www.gtexportal.org/home/). The RBP binding site data were retrieved from Postar2 (http://lulab.life.tsinghua.edu.cn/postar/). The miRNA binding site data were downloaded from targetScanHuman (http://www.targetscan.org/vert_72/).
