# Supplementary material for: Genetic variants associated mRNA stability in lung
Source: BMC Genomics. 2022 Mar 11;23:196. doi: 10.1186/s12864-022-08405-y (PMC8915503; doi:10.1186/s12864-022-08405-y)
Supplement: Supplementary file 3 — Additional file 3. Contains additional tables (Table S3, S4, S7, S8, and S9) and figures (Fig. S1 and S2) related to enrichment of RBP binding sites, enrichment of histone modifications using GARFIELD, and genic distribution of stQTLs/eQTLs after LD filtering. [file 12864_2022_8405_MOESM3_ESM.docx]

**Supplementary Information**

**Table S3.** RBP motifs of rs3167757 variant C.

| RBP | GenomicCoordinate | Motif | K-mer | Z-score | P-value |
| --- | --- | --- | --- | --- | --- |
| CUG-BP | chr21:40714482-40714478 | ugcug | ugcug | 2.92 | 2.E-03 |
| CUG-BP | chr21:40714479-40714475 | ugcug | uguug | 2.48 | 7.E-03 |
| HNRNPF | chr21:40714481-40714475 | gukgykg | gcuguug | 2.54 | 6.E-03 |
| MBNL1 | chr21:40714482-40714477 | ygcuky | ugcugu | 2.49 | 6.E-03 |
| MBNL1 | chr21:40714479-40714474 | ygcuky | uguuga | 1.68 | 5.E-02 |
| SFPQ | chr21:40714483-40714477 | kurrukk | gugcugu | 3.57 | 2.E-04 |
| TRA2B | chr21:40714485-40714478 | aaguguu | acgugcu | 2.47 | 7.E-03 |
| TRA2B | chr21:40714482-40714476 | aaguguu | ugcuguu | 1.81 | 4.E-02 |
| HNRNPL | chr21:40714472-40714478 | acacrav | acucaac | 3.25 | 6.E-04 |
| SRSF3 | chr21:40714474-40714478 | wcwwc | ucaac | 3.65 | 1.E-04 |
| SRSF3 | chr21:40714477-40714481 | wcwwc | acagc | 2.78 | 3.E-03 |
| YBX2 | chr21:40714476-40714482 | aacawcd | aacagca | 1.87 | 3.E-02 |

**Table S4.** The enrichment analysis of different histone modification peaks using GARFIELD. (A) Odds ratio (OR) and P-value of stQTLs and eQTLs after meta-analysis using Stouffer's method. (B) Odds ratio (OR) and P-value of stQTLs and eQTLs for each histone modification dataset.

**(A)**

| Type | OR_stQTL | metaP_stQTL | OR_eQTL | metaP_eQTL |
| --- | --- | --- | --- | --- |
| H2AFZ | 1.17 | 2.15E-15 | 1.17 | 5.48E-19 |
| H3K27ac | 1.30 | 5.04E-76 | 1.19 | 4.35E-43 |
| H3K27me3 | 0.88 | 5.60E-22 | 0.93 | 1.60E-10 |
| H3K36me3 | 1.53 | 1.41E-262 | 1.29 | 4.06E-114 |
| H3K4me1 | 1.17 | 2.77E-27 | 1.10 | 3.67E-12 |
| H3K4me2 | 1.22 | 3.47E-55 | 1.19 | 3.11E-56 |
| H3K4me3 | 1.25 | 3.10E-60 | 1.22 | 1.31E-61 |
| H3K79me2 | 1.35 | 7.00E-78 | 1.21 | 1.79E-41 |
| H3K9ac | 1.37 | 4.89E-125 | 1.28 | 2.99E-97 |
| H3K9me3 | 1.10 | 1.21E-01 | 1.02 | 7.60E-01 |
| H4K20me1 | 1.21 | 3.42E-19 | 1.07 | 2.25E-04 |

**(B)**

| Annotation | Type | OR_eQTL | FDR_eQTL | OR_stQTL | FDR_stQTL |
| --- | --- | --- | --- | --- | --- |
| H2AFZ_gm12878 | H2AFZ | 1.16 | 3.18E-07 | 1.19 | 2.11E-07 |
| H2AFZ_hepg2 | H2AFZ | 1.15 | 3.79E-05 | 1.12 | 2.60E-03 |
| H2AFZ_k562 | H2AFZ | 1.19 | 1.85E-10 | 1.20 | 7.20E-09 |
| H3K27ac_gm12878 | H3K27ac | 1.22 | 4.71E-12 | 1.41 | 8.60E-28 |
| H3K27ac_h1hesc | H3K27ac | 1.25 | 3.63E-10 | 1.33 | 1.60E-12 |
| H3K27ac_helas3 | H3K27ac | 1.09 | 5.80E-03 | 1.15 | 4.81E-05 |
| H3K27ac_hepg2 | H3K27ac | 1.23 | 3.69E-12 | 1.32 | 4.87E-17 |
| H3K27ac_huvec | H3K27ac | 1.06 | 4.21E-02 | 1.18 | 4.69E-07 |
| H3K27ac_k562 | H3K27ac | 1.30 | 1.02E-19 | 1.38 | 1.14E-22 |
| H3K27me3_gm12878 | H3K27me3 | 0.89 | 7.99E-03 | 0.80 | 6.17E-06 |
| H3K27me3_h1hesc | H3K27me3 | 1.02 | 5.75E-01 | 1.02 | 6.91E-01 |
| H3K27me3_helas3 | H3K27me3 | 0.87 | 3.47E-05 | 0.84 | 6.17E-06 |
| H3K27me3_hepg2 | H3K27me3 | 0.94 | 2.62E-02 | 0.89 | 2.12E-04 |
| H3K27me3_huvec | H3K27me3 | 1.04 | 1.38E-01 | 0.90 | 2.35E-03 |
| H3K27me3_k562 | H3K27me3 | 0.82 | 1.15E-15 | 0.80 | 5.45E-15 |
| H3K36me3_gm12878 | H3K36me3 | 1.29 | 5.60E-23 | 1.62 | 4.71E-64 |
| H3K36me3_h1hesc | H3K36me3 | 1.33 | 2.78E-18 | 1.47 | 1.04E-26 |
| H3K36me3_helas3 | H3K36me3 | 1.23 | 3.31E-13 | 1.43 | 6.59E-31 |
| H3K36me3_hepg2 | H3K36me3 | 1.30 | 8.17E-22 | 1.48 | 1.76E-39 |
| H3K36me3_huvec | H3K36me3 | 1.28 | 3.60E-21 | 1.53 | 8.29E-51 |
| H3K36me3_k562 | H3K36me3 | 1.32 | 8.08E-24 | 1.66 | 6.43E-64 |
| H3K4me1_gm12878 | H3K4me1 | 1.06 | 3.30E-02 | 1.19 | 6.15E-09 |
| H3K4me1_h1hesc | H3K4me1 | 1.01 | 7.94E-01 | 0.95 | 1.26E-01 |
| H3K4me1_huvec | H3K4me1 | 0.98 | 4.67E-01 | 1.09 | 2.77E-03 |
| H3K4me1_k562 | H3K4me1 | 1.35 | 1.78E-37 | 1.46 | 1.45E-45 |
| H3K4me2_gm12878 | H3K4me2 | 1.20 | 7.48E-12 | 1.32 | 4.14E-21 |
| H3K4me2_h1hesc | H3K4me2 | 1.20 | 8.07E-11 | 1.17 | 1.38E-06 |
| H3K4me2_helas3 | H3K4me2 | 1.15 | 2.39E-07 | 1.17 | 2.63E-07 |
| H3K4me2_hepg2 | H3K4me2 | 1.23 | 6.42E-16 | 1.19 | 3.24E-09 |
| H3K4me2_huvec | H3K4me2 | 1.10 | 4.10E-04 | 1.14 | 2.82E-05 |
| H3K4me2_k562 | H3K4me2 | 1.27 | 6.87E-18 | 1.32 | 2.03E-19 |
| H3K4me3_gm12878 | H3K4me3 | 1.26 | 2.00E-16 | 1.38 | 4.13E-25 |
| H3K4me3_h1hesc | H3K4me3 | 1.22 | 6.08E-10 | 1.17 | 1.14E-05 |
| H3K4me3_helas3 | H3K4me3 | 1.12 | 1.52E-04 | 1.12 | 4.62E-04 |
| H3K4me3_hepg2 | H3K4me3 | 1.26 | 1.59E-15 | 1.27 | 3.26E-13 |
| H3K4me3_huvec | H3K4me3 | 1.23 | 9.75E-11 | 1.24 | 1.12E-09 |
| H3K4me3_k562 | H3K4me3 | 1.26 | 1.55E-15 | 1.32 | 3.61E-18 |
| H3K79me2_gm12878 | H3K79me2 | 1.24 | 8.87E-14 | 1.47 | 6.20E-35 |
| H3K79me2_helas3 | H3K79me2 | 1.21 | 1.17E-11 | 1.29 | 1.56E-15 |
| H3K79me2_hepg2 | H3K79me2 | 1.17 | 5.39E-08 | 1.25 | 1.75E-11 |
| H3K79me2_k562 | H3K79me2 | 1.24 | 3.17E-13 | 1.39 | 9.91E-25 |
| H3K9ac_gm12878 | H3K9ac | 1.26 | 2.43E-15 | 1.46 | 6.12E-32 |
| H3K9ac_h1hesc | H3K9ac | 1.24 | 9.40E-11 | 1.41 | 3.55E-20 |
| H3K9ac_helas3 | H3K9ac | 1.28 | 2.78E-18 | 1.30 | 1.58E-16 |
| H3K9ac_hepg2 | H3K9ac | 1.31 | 3.88E-21 | 1.35 | 9.74E-21 |
| H3K9ac_huvec | H3K9ac | 1.13 | 5.01E-05 | 1.20 | 1.62E-07 |
| H3K9ac_k562 | H3K9ac | 1.43 | 7.56E-47 | 1.50 | 2.23E-46 |
| H3K9me3_gm12878 | H3K9me3 | 0.92 | 3.69E-02 | 1.05 | 3.04E-01 |
| H3K9me3_k562 | H3K9me3 | 1.12 | 8.33E-02 | 1.15 | 5.36E-02 |
| H4K20me1_gm12878 | H4K20me1 | 1.11 | 7.25E-02 | 1.59 | 3.91E-16 |
| H4K20me1_h1hesc | H4K20me1 | 1.06 | 2.12E-01 | 1.12 | 2.96E-02 |
| H4K20me1_helas3 | H4K20me1 | 1.20 | 2.88E-05 | 1.31 | 1.78E-08 |
| H4K20me1_hepg2 | H4K20me1 | 1.14 | 7.99E-04 | 1.22 | 4.46E-06 |
| H4K20me1_huvec | H4K20me1 | 0.87 | 4.63E-02 | 0.94 | 4.11E-01 |
| H4K20me1_k562 | H4K20me1 | 1.06 | 1.46E-01 | 1.10 | 2.49E-02 |

**Table S7.** Fisher's exact test between stQTLs in RBP binding sites and variant-gene pairs in RBP binding sites in each genic region of pre-mRNA.

|  | stQTL in RBPBS | variant-gene in RBPBS | ER | P-value |
| --- | --- | --- | --- | --- |
| 5'UTR | 941 | 43351 | 0.79 | > 0.1 |
| CDS | 4100 | 60250 | 2.47 | < 2e-308 |
| Intron | 8861 | 455602 | 0.71 | > 0.1 |
| 3'UTR | 2681 | 42113 | 2.31 | < 2e-308 |

**Table S8.** Fisher's exact test between stQTL and not stQTL on mature mRNA (two-sides).

| RBPs | stQTL | non-stQTL | ER | P-value | log2ER | FDR |
| --- | --- | --- | --- | --- | --- | --- |
| ELAVL1 | 332 | 23776 | 0.70 | 2.E-11 | -0.52 | 3.E-09 |
| SND1 | 64 | 1504 | 2.17 | 5.E-08 | 1.12 | 5.E-06 |
| YTHDC1 | 100 | 2850 | 1.80 | 1.E-07 | 0.84 | 7.E-06 |
| HNRNPC | 605 | 37443 | 0.81 | 2.E-07 | -0.31 | 1.E-05 |
| FMR1 | 80 | 6712 | 0.60 | 1.E-06 | -0.73 | 5.E-05 |
| DDX3X | 226 | 8348 | 1.39 | 5.E-06 | 0.47 | 1.E-04 |
| ATXN2 | 436 | 17911 | 1.25 | 1.E-05 | 0.33 | 3.E-04 |
| RPS3 | 91 | 2923 | 1.59 | 5.E-05 | 0.67 | 1.E-03 |
| UPF1 | 198 | 7533 | 1.35 | 8.E-05 | 0.43 | 2.E-03 |
| LARP4 | 55 | 1648 | 1.70 | 3.E-04 | 0.77 | 5.E-03 |
| FXR2 | 24 | 2278 | 0.53 | 1.E-03 | -0.90 | 2.E-02 |
| UCHL5 | 86 | 3067 | 1.43 | 2.E-03 | 0.52 | 2.E-02 |
| EFTUD2 | 103 | 3920 | 1.34 | 5.E-03 | 0.42 | 6.E-02 |
| RBM15B | 37 | 1157 | 1.63 | 6.E-03 | 0.70 | 7.E-02 |
| NOL12 | 18 | 453 | 2.02 | 6.E-03 | 1.02 | 7.E-02 |
| SRSF1 | 98 | 3748 | 1.33 | 7.E-03 | 0.42 | 7.E-02 |
| ZC3H7B | 85 | 5753 | 0.75 | 7.E-03 | -0.42 | 7.E-02 |
| EIF4G2 | 4 | 639 | 0.32 | 9.E-03 | -1.65 | 9.E-02 |
| HNRNPA2B1 | 3 | 21 | 7.27 | 1.E-02 | 2.86 | 1.E-01 |
| PRPF8 | 120 | 4846 | 1.26 | 1.E-02 | 0.34 | 1.E-01 |
| FXR1 | 2 | 440 | 0.23 | 1.E-02 | -2.11 | 1.E-01 |
| QKI | 27 | 852 | 1.61 | 2.E-02 | 0.69 | 1.E-01 |
| SRSF9 | 12 | 305 | 2.00 | 2.E-02 | 1.00 | 2.E-01 |
| SSB | 1 | 327 | 0.16 | 2.E-02 | -2.69 | 2.E-01 |
| EIF3B | 20 | 591 | 1.72 | 3.E-02 | 0.79 | 2.E-01 |
| RBM22 | 22 | 684 | 1.64 | 3.E-02 | 0.71 | 2.E-01 |
| PPIG | 100 | 4077 | 1.25 | 3.E-02 | 0.32 | 2.E-01 |
| HNRNPM | 19 | 1571 | 0.61 | 3.E-02 | -0.70 | 2.E-01 |
| PTBP1 | 48 | 3291 | 0.74 | 4.E-02 | -0.43 | 2.E-01 |
| METAP2 | 9 | 896 | 0.51 | 4.E-02 | -0.97 | 2.E-01 |
| HNRNPA1 | 28 | 2095 | 0.68 | 4.E-02 | -0.56 | 2.E-01 |
| RBM47 | 59 | 2273 | 1.32 | 4.E-02 | 0.40 | 2.E-01 |
| SMNDC1 | 20 | 628 | 1.62 | 4.E-02 | 0.70 | 2.E-01 |
| KHSRP | 16 | 1309 | 0.62 | 6.E-02 | -0.69 | 3.E-01 |
| PCBP2 | 30 | 2149 | 0.71 | 6.E-02 | -0.50 | 3.E-01 |
| CSTF2 | 206 | 11922 | 0.88 | 6.E-02 | -0.19 | 3.E-01 |
| SUB1 | 31 | 1109 | 1.42 | 7.E-02 | 0.51 | 3.E-01 |
| SF3A3 | 31 | 1129 | 1.40 | 7.E-02 | 0.48 | 3.E-01 |
| ZNF622 | 30 | 2121 | 0.72 | 7.E-02 | -0.48 | 3.E-01 |
| GRWD1 | 90 | 3782 | 1.21 | 8.E-02 | 0.28 | 3.E-01 |
| STAU1 | 14 | 429 | 1.66 | 8.E-02 | 0.73 | 3.E-01 |
| DDX42 | 0 | 170 | 0.00 | 8.E-02 |  | 3.E-01 |
| YTHDF1 | 51 | 2051 | 1.27 | 9.E-02 | 0.34 | 4.E-01 |
| FTO | 2 | 314 | 0.32 | 1.E-01 | -1.63 | 4.E-01 |
| DHX30 | 6 | 602 | 0.51 | 1.E-01 | -0.98 | 4.E-01 |
| SLTM | 30 | 1112 | 1.37 | 1.E-01 | 0.46 | 4.E-01 |
| FAM120A | 53 | 3387 | 0.79 | 1.E-01 | -0.33 | 4.E-01 |
| HNRNPD | 10 | 866 | 0.59 | 1.E-01 | -0.77 | 4.E-01 |
| NOP56 | 0 | 138 | 0.00 | 1.E-01 |  | 4.E-01 |
| MOV10 | 97 | 5780 | 0.85 | 1.E-01 | -0.23 | 4.E-01 |
| DDX55 | 45 | 1805 | 1.27 | 1.E-01 | 0.34 | 4.E-01 |
| DKC1 | 3 | 383 | 0.40 | 1.E-01 | -1.33 | 4.E-01 |
| YTHDF3 | 17 | 603 | 1.44 | 1.E-01 | 0.52 | 4.E-01 |
| CPSF7 | 205 | 9411 | 1.11 | 1.E-01 | 0.15 | 4.E-01 |
| TNRC6C | 1 | 7 | 7.27 | 1.E-01 | 2.86 | 4.E-01 |
| CELF2 | 27 | 1043 | 1.32 | 1.E-01 | 0.40 | 4.E-01 |
| FASTKD2 | 7 | 637 | 0.56 | 1.E-01 | -0.84 | 4.E-01 |
| NCBP2 | 45 | 1837 | 1.25 | 2.E-01 | 0.32 | 4.E-01 |
| IGF2BP3 | 78 | 4693 | 0.84 | 2.E-01 | -0.24 | 4.E-01 |
| YBX3 | 41 | 1661 | 1.26 | 2.E-01 | 0.33 | 4.E-01 |
| TNRC6A | 8 | 246 | 1.66 | 2.E-01 | 0.73 | 5.E-01 |
| IGF2BP2 | 66 | 4003 | 0.84 | 2.E-01 | -0.26 | 5.E-01 |
| HNRNPK | 16 | 1183 | 0.69 | 2.E-01 | -0.54 | 5.E-01 |
| KHDRBS1 | 3 | 71 | 2.15 | 2.E-01 | 1.10 | 5.E-01 |
| WDR33 | 54 | 2269 | 1.21 | 2.E-01 | 0.28 | 5.E-01 |
| RTCB | 23 | 894 | 1.31 | 2.E-01 | 0.39 | 5.E-01 |
| TIAL1 | 54 | 2295 | 1.20 | 2.E-01 | 0.26 | 5.E-01 |
| SF3B4 | 61 | 2630 | 1.18 | 2.E-01 | 0.24 | 5.E-01 |
| XPO5 | 6 | 534 | 0.57 | 2.E-01 | -0.81 | 5.E-01 |
| TARDBP | 327 | 17795 | 0.93 | 2.E-01 | -0.10 | 5.E-01 |
| NKRF | 10 | 770 | 0.66 | 2.E-01 | -0.60 | 6.E-01 |
| EIF3G | 24 | 949 | 1.29 | 2.E-01 | 0.36 | 6.E-01 |
| BCCIP | 2 | 47 | 2.17 | 2.E-01 | 1.11 | 6.E-01 |
| TIA1 | 42 | 1803 | 1.19 | 3.E-01 | 0.25 | 6.E-01 |
| LARP7 | 1 | 178 | 0.29 | 3.E-01 | -1.81 | 6.E-01 |
| AGGF1 | 18 | 700 | 1.31 | 3.E-01 | 0.39 | 6.E-01 |
| GTF2F1 | 20 | 1329 | 0.76 | 3.E-01 | -0.39 | 6.E-01 |
| RBM27 | 1 | 188 | 0.27 | 3.E-01 | -1.89 | 6.E-01 |
| YTHDF2 | 73 | 4235 | 0.88 | 3.E-01 | -0.19 | 6.E-01 |
| DDX6 | 39 | 1686 | 1.18 | 3.E-01 | 0.24 | 6.E-01 |
| SRSF7 | 3 | 315 | 0.48 | 3.E-01 | -1.05 | 6.E-01 |
| DICER1 | 6 | 205 | 1.49 | 3.E-01 | 0.57 | 6.E-01 |
| CPSF2 | 6 | 211 | 1.45 | 3.E-01 | 0.53 | 7.E-01 |
| XRCC6 | 9 | 337 | 1.36 | 3.E-01 | 0.44 | 7.E-01 |
| U2AF1 | 4 | 359 | 0.57 | 3.E-01 | -0.82 | 7.E-01 |
| LSM11 | 28 | 1181 | 1.21 | 3.E-01 | 0.27 | 7.E-01 |
| GRSF1 | 10 | 371 | 1.37 | 3.E-01 | 0.46 | 7.E-01 |
| SUGP2 | 7 | 540 | 0.66 | 3.E-01 | -0.60 | 7.E-01 |
| HNRNPH1 | 4 | 142 | 1.43 | 4.E-01 | 0.52 | 7.E-01 |
| BUD13 | 90 | 4165 | 1.10 | 4.E-01 | 0.14 | 7.E-01 |
| RBM15 | 49 | 2852 | 0.87 | 4.E-01 | -0.20 | 7.E-01 |
| DIS3L2 | 1 | 154 | 0.33 | 4.E-01 | -1.60 | 7.E-01 |
| SF3B1 | 22 | 1372 | 0.82 | 4.E-01 | -0.29 | 7.E-01 |
| NPM1 | 1 | 24 | 2.12 | 4.E-01 | 1.08 | 7.E-01 |
| U2AF2 | 92 | 5154 | 0.91 | 4.E-01 | -0.14 | 7.E-01 |
| FIP1L1 | 155 | 8458 | 0.93 | 4.E-01 | -0.10 | 7.E-01 |
| TAF15 | 17 | 705 | 1.23 | 4.E-01 | 0.30 | 7.E-01 |
| FKBP4 | 4 | 350 | 0.58 | 4.E-01 | -0.78 | 8.E-01 |
| AARS | 8 | 560 | 0.73 | 4.E-01 | -0.46 | 8.E-01 |
| CNBP | 3 | 98 | 1.56 | 4.E-01 | 0.64 | 8.E-01 |
| EIF3H | 25 | 1519 | 0.84 | 5.E-01 | -0.26 | 8.E-01 |
| CDC40 | 5 | 386 | 0.66 | 5.E-01 | -0.60 | 8.E-01 |
| MBNL2 | 5 | 400 | 0.64 | 5.E-01 | -0.65 | 8.E-01 |
| CPSF3 | 35 | 1592 | 1.12 | 5.E-01 | 0.16 | 8.E-01 |
| DGCR8 | 15 | 635 | 1.20 | 5.E-01 | 0.27 | 8.E-01 |
| GNL3 | 1 | 36 | 1.41 | 5.E-01 | 0.50 | 8.E-01 |
| CSTF2T | 252 | 12323 | 1.04 | 5.E-01 | 0.06 | 8.E-01 |
| DROSHA | 22 | 1299 | 0.86 | 5.E-01 | -0.22 | 9.E-01 |
| EWSR1 | 51 | 2386 | 1.09 | 6.E-01 | 0.12 | 9.E-01 |
| TARBP2 | 4 | 159 | 1.28 | 6.E-01 | 0.36 | 9.E-01 |
| SFPQ | 5 | 357 | 0.71 | 6.E-01 | -0.49 | 9.E-01 |
| IGF2BP1 | 93 | 5048 | 0.94 | 6.E-01 | -0.09 | 9.E-01 |
| XRN2 | 27 | 1561 | 0.88 | 6.E-01 | -0.19 | 9.E-01 |
| CAPRIN1 | 53 | 2948 | 0.91 | 6.E-01 | -0.13 | 9.E-01 |
| SAFB2 | 2 | 197 | 0.52 | 6.E-01 | -0.95 | 9.E-01 |
| CPSF1 | 21 | 1230 | 0.87 | 6.E-01 | -0.20 | 9.E-01 |
| DDX24 | 55 | 2616 | 1.07 | 6.E-01 | 0.10 | 9.E-01 |
| TBRG4 | 15 | 891 | 0.86 | 6.E-01 | -0.22 | 9.E-01 |
| HNRNPF | 0 | 60 | 0.00 | 6.E-01 |  | 9.E-01 |
| RBM10 | 0 | 66 | 0.00 | 6.E-01 |  | 9.E-01 |
| ZRANB2 | 12 | 548 | 1.11 | 6.E-01 | 0.16 | 9.E-01 |
| CPSF4 | 19 | 1098 | 0.88 | 7.E-01 | -0.18 | 9.E-01 |
| FBL | 6 | 273 | 1.12 | 7.E-01 | 0.16 | 9.E-01 |
| RPS5 | 14 | 643 | 1.11 | 7.E-01 | 0.15 | 9.E-01 |
| YTHDC2 | 2 | 86 | 1.18 | 7.E-01 | 0.24 | 9.E-01 |
| RBM5 | 5 | 346 | 0.73 | 7.E-01 | -0.44 | 9.E-01 |
| RBFOX2 | 32 | 1533 | 1.06 | 7.E-01 | 0.09 | 1.E+00 |
| NUDT21 | 166 | 8233 | 1.03 | 7.E-01 | 0.04 | 1.E+00 |
| LIN28B | 203 | 10609 | 0.97 | 7.E-01 | -0.04 | 1.E+00 |
| CPSF6 | 134 | 7071 | 0.96 | 7.E-01 | -0.05 | 1.E+00 |
| RBPMS | 1 | 118 | 0.43 | 7.E-01 | -1.21 | 1.E+00 |
| EIF3A | 11 | 517 | 1.08 | 8.E-01 | 0.11 | 1.E+00 |
| METTL3 | 3 | 141 | 1.08 | 8.E-01 | 0.11 | 1.E+00 |
| ILF3 | 12 | 566 | 1.08 | 8.E-01 | 0.11 | 1.E+00 |
| FUBP3 | 13 | 626 | 1.06 | 8.E-01 | 0.08 | 1.E+00 |
| GEMIN5 | 42 | 2080 | 1.03 | 8.E-01 | 0.04 | 1.E+00 |
| EIF3D | 19 | 937 | 1.03 | 8.E-01 | 0.04 | 1.E+00 |
| TROVE2 | 4 | 258 | 0.79 | 8.E-01 | -0.34 | 1.E+00 |
| FUS | 91 | 4768 | 0.97 | 8.E-01 | -0.04 | 1.E+00 |
| SRRM4 | 25 | 1354 | 0.94 | 8.E-01 | -0.09 | 1.E+00 |
| NCBP3 | 14 | 783 | 0.91 | 9.E-01 | -0.14 | 1.E+00 |
| LIN28A | 29 | 1448 | 1.02 | 9.E-01 | 0.03 | 1.E+00 |
| PUM2 | 37 | 1856 | 1.01 | 9.E-01 | 0.02 | 1.E+00 |
| ALKBH5 | 8 | 408 | 1.00 | 1.E+00 | 0.00 | 1.E+00 |
| AKAP8L | 11 | 562 | 1.00 | 1.E+00 | -0.01 | 1.E+00 |
| EIF4A3 | 14 | 716 | 0.99 | 1.E+00 | -0.01 | 1.E+00 |
| DDX59 | 11 | 568 | 0.99 | 1.E+00 | -0.02 | 1.E+00 |
| NONO | 25 | 1304 | 0.98 | 1.E+00 | -0.04 | 1.E+00 |
| SERBP1 | 5 | 263 | 0.97 | 1.E+00 | -0.05 | 1.E+00 |
| GPKOW | 9 | 474 | 0.97 | 1.E+00 | -0.05 | 1.E+00 |
| HNRNPU | 8 | 434 | 0.94 | 1.E+00 | -0.09 | 1.E+00 |
| NOP58 | 4 | 218 | 0.93 | 1.E+00 | -0.10 | 1.E+00 |
| SUPV3L1 | 6 | 333 | 0.92 | 1.E+00 | -0.13 | 1.E+00 |
| TRA2A | 9 | 508 | 0.90 | 1.E+00 | -0.15 | 1.E+00 |
| MTPAP | 5 | 302 | 0.84 | 1.E+00 | -0.25 | 1.E+00 |
| ALKBH1 | 1 | 61 | 0.83 | 1.E+00 | -0.26 | 1.E+00 |
| YWHAG | 2 | 129 | 0.79 | 1.E+00 | -0.34 | 1.E+00 |
| METTL14 | 1 | 65 | 0.78 | 1.E+00 | -0.35 | 1.E+00 |
| WTAP | 1 | 66 | 0.77 | 1.E+00 | -0.38 | 1.E+00 |
| HNRNPUL1 | 2 | 135 | 0.75 | 1.E+00 | -0.41 | 1.E+00 |
| EZH2 | 0 | 1 | 0.00 | 1.E+00 |  | 1.E+00 |
| RPS11 | 0 | 48 | 0.00 | 1.E+00 |  | 1.E+00 |
| AUH | 0 | 20 | 0.00 | 1.E+00 |  | 1.E+00 |
| TNRC6B | 0 | 7 | 0.00 | 1.E+00 |  | 1.E+00 |
| SBDS | 0 | 9 | 0.00 | 1.E+00 |  | 1.E+00 |
| PUS1 | 0 | 40 | 0.00 | 1.E+00 |  | 1.E+00 |
| SLBP | 0 | 22 | 0.00 | 1.E+00 |  | 1.E+00 |
| PRKRA | 0 | 23 | 0.00 | 1.E+00 |  | 1.E+00 |
| PPIL4 | 0 | 40 | 0.00 | 1.E+00 |  | 1.E+00 |
| NSUN2 | 0 | 14 | 0.00 | 1.E+00 |  | 1.E+00 |
| HLTF | 0 | 27 | 0.00 | 1.E+00 |  | 1.E+00 |

**Table S9.** Fisher's exact test between eQTL and not eQTL on mature mRNA (two-sides).

| RBPs | eQTL | non-eQTL | ER | P-value | log2ER | FDR |
| --- | --- | --- | --- | --- | --- | --- |
| DDX3X | 250 | 8331 | 1.69 | 6.E-14 | 0.76 | 1.E-11 |
| ELAVL1 | 298 | 23808 | 0.68 | 1.E-11 | -0.55 | 1.E-09 |
| HNRNPC | 523 | 37536 | 0.76 | 3.E-10 | -0.40 | 1.E-08 |
| SND1 | 58 | 1509 | 2.15 | 4.E-07 | 1.10 | 2.E-05 |
| FMR1 | 75 | 6718 | 0.62 | 1.E-05 | -0.70 | 3.E-04 |
| NCBP2 | 60 | 1819 | 1.84 | 2.E-05 | 0.88 | 6.E-04 |
| AGGF1 | 30 | 689 | 2.43 | 2.E-05 | 1.28 | 6.E-04 |
| ZC3H7B | 67 | 5767 | 0.64 | 2.E-04 | -0.64 | 3.E-03 |
| GRWD1 | 101 | 3773 | 1.50 | 2.E-04 | 0.58 | 3.E-03 |
| MOV10 | 68 | 5806 | 0.65 | 2.E-04 | -0.63 | 3.E-03 |
| UCHL5 | 84 | 3069 | 1.53 | 3.E-04 | 0.61 | 5.E-03 |
| EFTUD2 | 102 | 3921 | 1.45 | 5.E-04 | 0.54 | 7.E-03 |
| IGF2BP2 | 44 | 4026 | 0.60 | 5.E-04 | -0.73 | 7.E-03 |
| SLTM | 37 | 1107 | 1.86 | 6.E-04 | 0.90 | 7.E-03 |
| DDX24 | 72 | 2598 | 1.55 | 7.E-04 | 0.63 | 7.E-03 |
| HNRNPA2B1 | 4 | 20 | 11.12 | 8.E-04 | 3.48 | 8.E-03 |
| YTHDC1 | 76 | 2870 | 1.48 | 2.E-03 | 0.56 | 2.E-02 |
| IGF2BP3 | 57 | 4715 | 0.67 | 2.E-03 | -0.58 | 2.E-02 |
| LSM11 | 37 | 1174 | 1.76 | 2.E-03 | 0.81 | 2.E-02 |
| LARP4 | 48 | 1654 | 1.62 | 2.E-03 | 0.69 | 2.E-02 |
| RPS3 | 76 | 2938 | 1.44 | 3.E-03 | 0.53 | 2.E-02 |
| PPIG | 100 | 4080 | 1.37 | 3.E-03 | 0.45 | 2.E-02 |
| FXR2 | 23 | 2278 | 0.56 | 3.E-03 | -0.84 | 2.E-02 |
| SMNDC1 | 22 | 626 | 1.96 | 4.E-03 | 0.97 | 3.E-02 |
| HNRNPH1 | 8 | 138 | 3.23 | 5.E-03 | 1.69 | 3.E-02 |
| IGF2BP1 | 65 | 5077 | 0.71 | 5.E-03 | -0.50 | 3.E-02 |
| PRPF8 | 115 | 4849 | 1.32 | 5.E-03 | 0.40 | 3.E-02 |
| CDC40 | 15 | 377 | 2.21 | 6.E-03 | 1.15 | 3.E-02 |
| LIN28B | 155 | 10652 | 0.80 | 7.E-03 | -0.31 | 4.E-02 |
| TBRG4 | 6 | 900 | 0.37 | 7.E-03 | -1.43 | 4.E-02 |
| HNRNPM | 15 | 1574 | 0.53 | 1.E-02 | -0.92 | 5.E-02 |
| HNRNPD | 6 | 869 | 0.38 | 1.E-02 | -1.38 | 5.E-02 |
| BUD13 | 98 | 4155 | 1.32 | 1.E-02 | 0.40 | 5.E-02 |
| RPS5 | 21 | 635 | 1.84 | 1.E-02 | 0.88 | 5.E-02 |
| SRSF9 | 12 | 305 | 2.19 | 2.E-02 | 1.13 | 7.E-02 |
| RBFOX2 | 41 | 1526 | 1.50 | 2.E-02 | 0.58 | 7.E-02 |
| GEMIN5 | 52 | 2070 | 1.40 | 2.E-02 | 0.48 | 9.E-02 |
| SRSF1 | 87 | 3758 | 1.29 | 2.E-02 | 0.37 | 1.E-01 |
| YWHAG | 6 | 125 | 2.67 | 3.E-02 | 1.42 | 1.E-01 |
| ATXN2 | 362 | 17988 | 1.13 | 3.E-02 | 0.17 | 1.E-01 |
| NOP58 | 0 | 222 | 0.00 | 4.E-02 |  | 2.E-01 |
| RBM22 | 20 | 686 | 1.62 | 4.E-02 | 0.70 | 2.E-01 |
| GTF2F1 | 34 | 1316 | 1.44 | 5.E-02 | 0.52 | 2.E-01 |
| TNRC6A | 9 | 247 | 2.03 | 5.E-02 | 1.02 | 2.E-01 |
| SRSF7 | 1 | 317 | 0.18 | 5.E-02 | -2.51 | 2.E-01 |
| CPSF4 | 11 | 1106 | 0.55 | 5.E-02 | -0.86 | 2.E-01 |
| SERBP1 | 9 | 259 | 1.93 | 6.E-02 | 0.95 | 2.E-01 |
| EIF4A3 | 20 | 710 | 1.57 | 6.E-02 | 0.65 | 2.E-01 |
| RBM47 | 53 | 2277 | 1.30 | 7.E-02 | 0.37 | 2.E-01 |
| DDX55 | 43 | 1808 | 1.32 | 8.E-02 | 0.41 | 3.E-01 |
| GPKOW | 14 | 469 | 1.66 | 8.E-02 | 0.73 | 3.E-01 |
| HLTF | 2 | 25 | 4.45 | 8.E-02 | 2.15 | 3.E-01 |
| CPSF1 | 14 | 1236 | 0.63 | 8.E-02 | -0.67 | 3.E-01 |
| SAFB2 | 7 | 191 | 2.04 | 9.E-02 | 1.03 | 3.E-01 |
| RBM15B | 29 | 1167 | 1.38 | 1.E-01 | 0.47 | 3.E-01 |
| FXR1 | 3 | 439 | 0.38 | 1.E-01 | -1.40 | 3.E-01 |
| XRN2 | 37 | 1549 | 1.33 | 1.E-01 | 0.41 | 3.E-01 |
| YTHDF2 | 62 | 4245 | 0.81 | 1.E-01 | -0.30 | 3.E-01 |
| GRSF1 | 11 | 370 | 1.65 | 1.E-01 | 0.73 | 3.E-01 |
| UPF1 | 155 | 7587 | 1.14 | 1.E-01 | 0.19 | 3.E-01 |
| CSTF2 | 192 | 11940 | 0.89 | 1.E-01 | -0.17 | 3.E-01 |
| TNRC6C | 1 | 7 | 7.94 | 1.E-01 | 2.99 | 4.E-01 |
| FAM120A | 49 | 3391 | 0.80 | 1.E-01 | -0.32 | 4.E-01 |
| SSB | 2 | 326 | 0.34 | 1.E-01 | -1.55 | 4.E-01 |
| GNL3 | 2 | 35 | 3.18 | 1.E-01 | 1.67 | 4.E-01 |
| TROVE2 | 8 | 254 | 1.75 | 1.E-01 | 0.81 | 4.E-01 |
| SRRM4 | 17 | 1364 | 0.69 | 2.E-01 | -0.53 | 4.E-01 |
| TIA1 | 41 | 1805 | 1.26 | 2.E-01 | 0.34 | 4.E-01 |
| PUS1 | 2 | 38 | 2.93 | 2.E-01 | 1.55 | 4.E-01 |
| METAP2 | 10 | 895 | 0.62 | 2.E-01 | -0.69 | 4.E-01 |
| SF3B4 | 57 | 2634 | 1.20 | 2.E-01 | 0.27 | 4.E-01 |
| TARDBP | 296 | 17824 | 0.92 | 2.E-01 | -0.12 | 4.E-01 |
| DROSHA | 30 | 1291 | 1.29 | 2.E-01 | 0.37 | 4.E-01 |
| DKC1 | 3 | 383 | 0.44 | 2.E-01 | -1.20 | 4.E-01 |
| EIF3D | 11 | 943 | 0.65 | 2.E-01 | -0.63 | 4.E-01 |
| WDR33 | 32 | 2289 | 0.78 | 2.E-01 | -0.37 | 4.E-01 |
| NOP56 | 0 | 138 | 0.00 | 2.E-01 |  | 4.E-01 |
| FTO | 2 | 313 | 0.36 | 2.E-01 | -1.49 | 4.E-01 |
| ZRANB2 | 14 | 546 | 1.43 | 2.E-01 | 0.51 | 4.E-01 |
| BCCIP | 2 | 47 | 2.37 | 2.E-01 | 1.24 | 5.E-01 |
| XRCC6 | 9 | 337 | 1.49 | 2.E-01 | 0.57 | 5.E-01 |
| PUM2 | 26 | 1869 | 0.77 | 2.E-01 | -0.37 | 5.E-01 |
| EIF4G2 | 7 | 636 | 0.61 | 2.E-01 | -0.71 | 5.E-01 |
| FBL | 2 | 277 | 0.40 | 3.E-01 | -1.32 | 5.E-01 |
| PTBP1 | 50 | 3290 | 0.84 | 3.E-01 | -0.25 | 5.E-01 |
| ILF3 | 6 | 572 | 0.58 | 3.E-01 | -0.78 | 5.E-01 |
| CNBP | 3 | 98 | 1.70 | 3.E-01 | 0.77 | 5.E-01 |
| RBM15 | 59 | 2844 | 1.15 | 3.E-01 | 0.21 | 6.E-01 |
| CPSF7 | 156 | 9459 | 0.91 | 3.E-01 | -0.13 | 6.E-01 |
| NONO | 18 | 1311 | 0.76 | 3.E-01 | -0.39 | 6.E-01 |
| CAPRIN1 | 45 | 2955 | 0.85 | 3.E-01 | -0.24 | 6.E-01 |
| HNRNPUL1 | 4 | 133 | 1.67 | 3.E-01 | 0.74 | 6.E-01 |
| SUGP2 | 6 | 540 | 0.62 | 3.E-01 | -0.70 | 6.E-01 |
| EIF3G | 21 | 953 | 1.23 | 3.E-01 | 0.29 | 6.E-01 |
| EIF3H | 32 | 1512 | 1.18 | 3.E-01 | 0.24 | 6.E-01 |
| PRKRA | 1 | 22 | 2.53 | 3.E-01 | 1.34 | 6.E-01 |
| DDX59 | 13 | 566 | 1.28 | 3.E-01 | 0.35 | 6.E-01 |
| QKI | 19 | 859 | 1.23 | 4.E-01 | 0.30 | 6.E-01 |
| U2AF2 | 101 | 5148 | 1.09 | 4.E-01 | 0.13 | 6.E-01 |
| KHDRBS1 | 2 | 72 | 1.54 | 4.E-01 | 0.63 | 6.E-01 |
| NOL12 | 11 | 461 | 1.33 | 4.E-01 | 0.41 | 6.E-01 |
| SUPV3L1 | 8 | 330 | 1.35 | 4.E-01 | 0.43 | 7.E-01 |
| DDX6 | 35 | 1691 | 1.15 | 4.E-01 | 0.20 | 7.E-01 |
| YTHDC2 | 0 | 88 | 0.00 | 4.E-01 |  | 7.E-01 |
| CPSF6 | 118 | 7090 | 0.92 | 4.E-01 | -0.11 | 7.E-01 |
| U2AF1 | 4 | 359 | 0.62 | 4.E-01 | -0.69 | 7.E-01 |
| SF3A3 | 24 | 1136 | 1.18 | 4.E-01 | 0.23 | 7.E-01 |
| EWSR1 | 48 | 2387 | 1.12 | 4.E-01 | 0.16 | 7.E-01 |
| FUBP3 | 8 | 630 | 0.71 | 4.E-01 | -0.50 | 7.E-01 |
| FASTKD2 | 14 | 630 | 1.24 | 5.E-01 | 0.31 | 7.E-01 |
| ZNF622 | 33 | 2117 | 0.87 | 5.E-01 | -0.21 | 7.E-01 |
| STAU1 | 5 | 438 | 0.63 | 5.E-01 | -0.66 | 7.E-01 |
| SF3B1 | 28 | 1365 | 1.14 | 5.E-01 | 0.19 | 7.E-01 |
| CSTF2T | 232 | 12345 | 1.05 | 5.E-01 | 0.07 | 7.E-01 |
| HNRNPA1 | 33 | 2090 | 0.88 | 5.E-01 | -0.19 | 8.E-01 |
| PPIL4 | 1 | 39 | 1.43 | 5.E-01 | 0.51 | 8.E-01 |
| YBX3 | 26 | 1675 | 0.86 | 5.E-01 | -0.21 | 8.E-01 |
| AKAP8L | 12 | 561 | 1.19 | 5.E-01 | 0.25 | 8.E-01 |
| DHX30 | 8 | 600 | 0.74 | 5.E-01 | -0.43 | 8.E-01 |
| TARBP2 | 4 | 159 | 1.40 | 5.E-01 | 0.48 | 8.E-01 |
| RPS11 | 1 | 47 | 1.18 | 6.E-01 | 0.24 | 8.E-01 |
| RBM27 | 4 | 185 | 1.20 | 6.E-01 | 0.27 | 8.E-01 |
| HNRNPK | 18 | 1182 | 0.85 | 6.E-01 | -0.24 | 8.E-01 |
| HNRNPU | 9 | 433 | 1.16 | 6.E-01 | 0.21 | 8.E-01 |
| KHSRP | 20 | 1305 | 0.85 | 6.E-01 | -0.23 | 8.E-01 |
| YTHDF1 | 40 | 2062 | 1.08 | 6.E-01 | 0.11 | 8.E-01 |
| HNRNPF | 0 | 60 | 0.00 | 6.E-01 |  | 8.E-01 |
| AARS | 8 | 560 | 0.79 | 6.E-01 | -0.33 | 8.E-01 |
| METTL14 | 0 | 66 | 0.00 | 6.E-01 |  | 8.E-01 |
| RBM10 | 0 | 66 | 0.00 | 6.E-01 |  | 8.E-01 |
| SUB1 | 22 | 1114 | 1.10 | 7.E-01 | 0.14 | 8.E-01 |
| NUDT21 | 153 | 8243 | 1.03 | 7.E-01 | 0.05 | 9.E-01 |
| ALKBH5 | 8 | 408 | 1.09 | 7.E-01 | 0.12 | 9.E-01 |
| RBPMS | 1 | 118 | 0.47 | 7.E-01 | -1.09 | 9.E-01 |
| FIP1L1 | 156 | 8468 | 1.02 | 7.E-01 | 0.04 | 9.E-01 |
| EIF3A | 10 | 520 | 1.07 | 7.E-01 | 0.10 | 9.E-01 |
| LARP7 | 2 | 177 | 0.63 | 8.E-01 | -0.67 | 1.E+00 |
| CPSF3 | 30 | 1597 | 1.04 | 8.E-01 | 0.06 | 1.E+00 |
| NCBP3 | 15 | 781 | 1.07 | 8.E-01 | 0.09 | 1.E+00 |
| DICER1 | 4 | 207 | 1.07 | 8.E-01 | 0.10 | 1.E+00 |
| CPSF2 | 4 | 213 | 1.04 | 8.E-01 | 0.06 | 1.E+00 |
| TIAL1 | 43 | 2307 | 1.04 | 8.E-01 | 0.05 | 1.E+00 |
| CELF2 | 17 | 1052 | 0.90 | 8.E-01 | -0.16 | 1.E+00 |
| SFPQ | 5 | 357 | 0.78 | 8.E-01 | -0.36 | 1.E+00 |
| MBNL2 | 6 | 399 | 0.84 | 8.E-01 | -0.26 | 1.E+00 |
| TRA2A | 8 | 509 | 0.87 | 9.E-01 | -0.20 | 1.E+00 |
| PCBP2 | 37 | 2143 | 0.96 | 9.E-01 | -0.06 | 1.E+00 |
| EIF3B | 11 | 600 | 1.02 | 9.E-01 | 0.03 | 1.E+00 |
| DGCR8 | 12 | 638 | 1.05 | 9.E-01 | 0.06 | 1.E+00 |
| NKRF | 14 | 766 | 1.02 | 9.E-01 | 0.02 | 1.E+00 |
| RTCB | 15 | 902 | 0.92 | 9.E-01 | -0.11 | 1.E+00 |
| LIN28A | 25 | 1449 | 0.96 | 9.E-01 | -0.06 | 1.E+00 |
| DDX42 | 3 | 167 | 1.00 | 1.E+00 | 0.00 | 1.E+00 |
| FUS | 85 | 4774 | 0.99 | 1.E+00 | -0.02 | 1.E+00 |
| RBM5 | 6 | 345 | 0.97 | 1.E+00 | -0.05 | 1.E+00 |
| FKBP4 | 6 | 348 | 0.96 | 1.E+00 | -0.06 | 1.E+00 |
| XPO5 | 9 | 531 | 0.94 | 1.E+00 | -0.09 | 1.E+00 |
| TAF15 | 12 | 709 | 0.94 | 1.E+00 | -0.09 | 1.E+00 |
| MTPAP | 5 | 302 | 0.92 | 1.E+00 | -0.12 | 1.E+00 |
| YTHDF3 | 10 | 608 | 0.91 | 1.E+00 | -0.13 | 1.E+00 |
| ALKBH1 | 1 | 61 | 0.91 | 1.E+00 | -0.13 | 1.E+00 |
| WTAP | 1 | 66 | 0.84 | 1.E+00 | -0.25 | 1.E+00 |
| METTL3 | 2 | 142 | 0.78 | 1.E+00 | -0.35 | 1.E+00 |
| DIS3L2 | 2 | 154 | 0.72 | 1.E+00 | -0.47 | 1.E+00 |
| SLBP | 0 | 22 | 0.00 | 1.E+00 |  | 1.E+00 |
| AUH | 0 | 20 | 0.00 | 1.E+00 |  | 1.E+00 |
| NSUN2 | 0 | 14 | 0.00 | 1.E+00 |  | 1.E+00 |
| NPM1 | 0 | 25 | 0.00 | 1.E+00 |  | 1.E+00 |
| EZH2 | 0 | 1 | 0.00 | 1.E+00 |  | 1.E+00 |
| SBDS | 0 | 9 | 0.00 | 1.E+00 |  | 1.E+00 |
| TNRC6B | 0 | 7 | 0.00 | 1.E+00 |  | 1.E+00 |


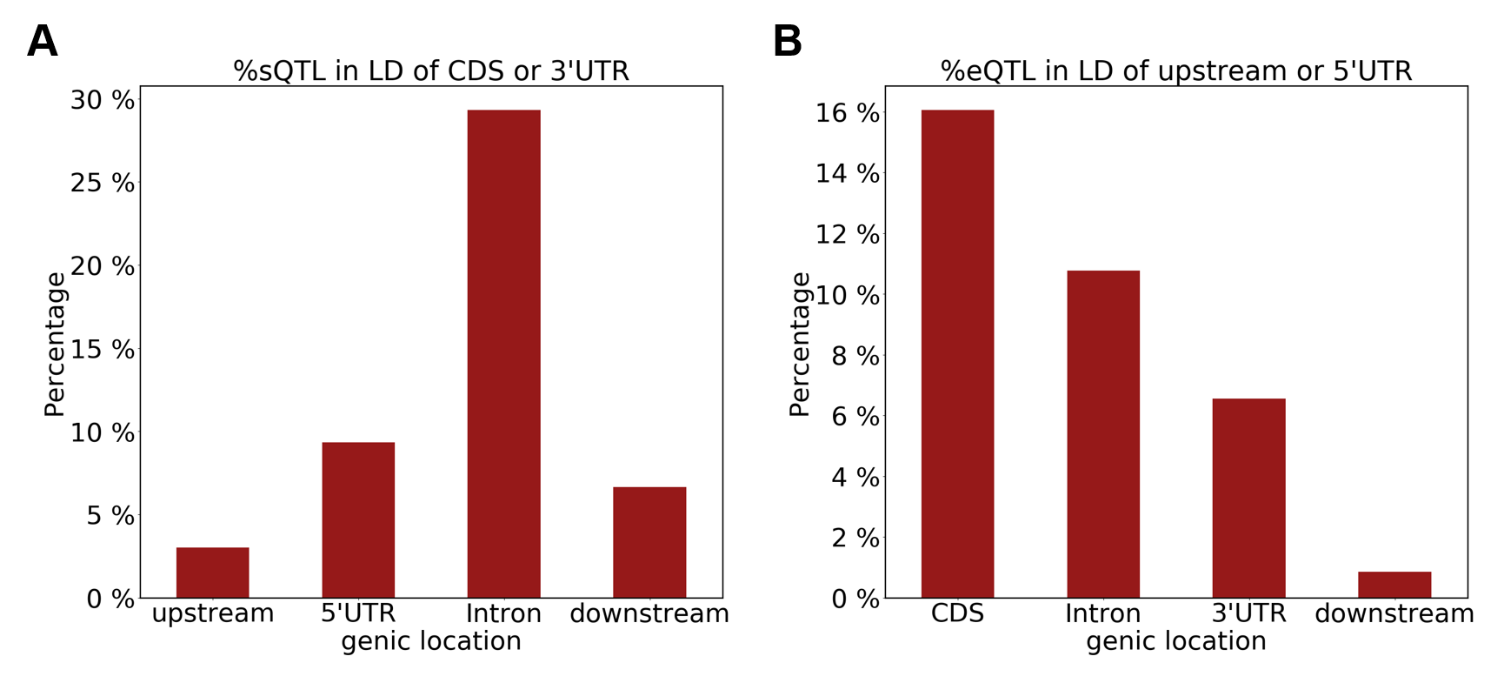


**Fig S1. There is a considerable part of stQTLs/eQTLs have strong LD (r^2^ > 0.9) with stQTLs/eQTLs in other regions.** (A) The proportion of stQTLs in intron, 5’UTR, downstream, and upstream regions that have a strong LD with stQTLs in CDS or 3’UTR. (B) The proportion of eQTLs in CDS, intron, 3’UTR, and downstream regions that have a strong LD with eQTLs in 5’UTR or upstream regions.


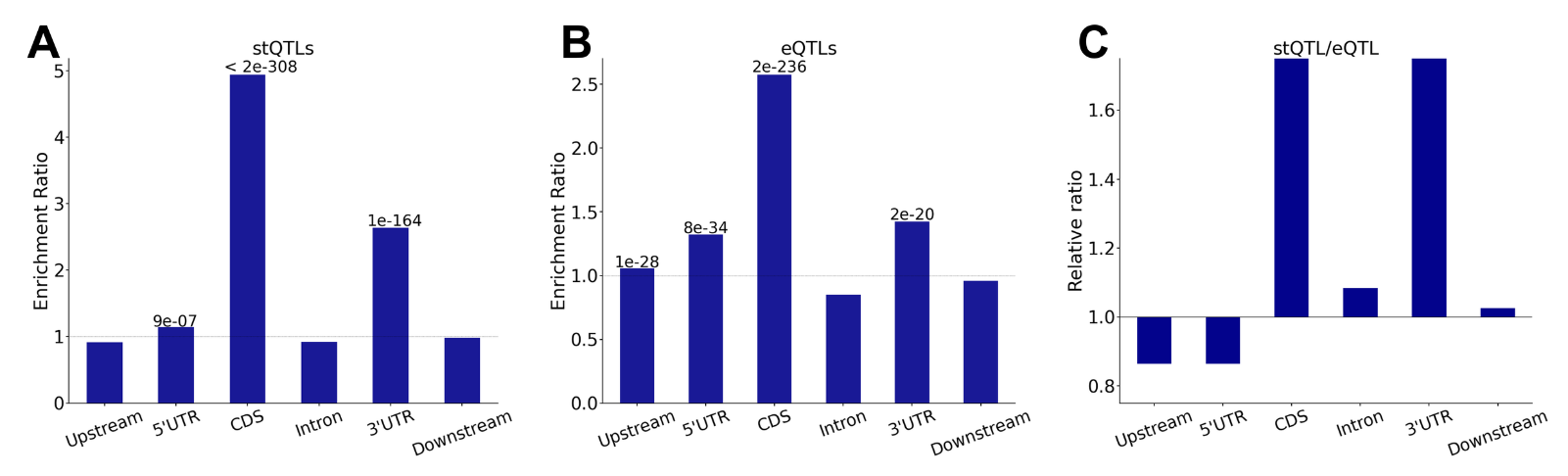


**Fig S2. There are biased distributions in different genic regions of eQTLs and stQTLs after LD filtering that selected the most significant genetic variant as the representative stQTL/eQTL in each LD blocks (r2 > 0.9).** (A) The enrichment ratio in different genic locations of stQTLs after LD filtering. (B) The enrichment ratio in different genic locations of eQTLs after LD filtering. (C) The relative proportion of enrichment ratio in different genic locations between stQTLs and eQTLs after LD filtering.
